# Quantifying trace element and isotope fluxes at the ocean–sediment boundary: a review

**DOI:** 10.1098/rsta.2016.0246

**Published:** 2016-11-28

**Authors:** William B. Homoky, Thomas Weber, William M. Berelson, Tim M. Conway, Gideon M. Henderson, Marco van Hulten, Catherine Jeandel, Silke Severmann, Alessandro Tagliabue

**Affiliations:** 1 Department of Earth Sciences, University of Oxford, South Parks Road, Oxford OX1 3AN, UK; 2 School of Oceanography, University of Washington, 1503 NE Boat Street, Seattle, WA 98105, USA; 3 Department of Earth Sciences, University of Southern California, Los Angeles, CA 90089, USA; 4 Department of Earth Sciences, ETH Zürich, Clausiusstrasse 25, 8092 Zürich, Switzerland; 5 College of Marine Science, University of South Florida, St Petersburg, FL 33701, USA; 6 Laboratoire des Sciences du Climat et de l'Environnement (LSCE), IPSL, CEA–Orme des Merisiers, 91191 Gif-sur-Yvette, France; 7 Laboratoire d'Etudes en Géophysique et Océanographie Spatiales (LEGOS), 14 Avenue Edouard Belin, 31400 Toulouse, France; 8 Department of Marine and Coastal Sciences, Rutgers University, 71 Dudley Road, New Brunswick, NJ 08901, USA; 9 School of Environmental Sciences, University of Liverpool, Jane Herdman Building, Liverpool L69 3GP, UK

**Keywords:** ocean, sediment, trace element, isotope, benthic boundary layer, GEOTRACES

## Abstract

Quantifying fluxes of trace elements and their isotopes (TEIs) at the ocean's sediment–water boundary is a pre-eminent challenge to understand their role in the present, past and future ocean. There are multiple processes that drive the uptake and release of TEIs, and properties that determine their rates are unevenly distributed (e.g. sediment composition, redox conditions and (bio)physical dynamics). These factors complicate our efforts to find, measure and extrapolate TEI fluxes across ocean basins. GEOTRACES observations are unveiling the oceanic distributions of many TEIs for the first time. These data evidence the influence of the sediment–water boundary on many TEI cycles, and underline the fact that our knowledge of the source–sink fluxes that sustain oceanic distributions is largely missing. Present flux measurements provide low spatial coverage and only part of the empirical basis needed to predict TEI flux variations. Many of the advances and present challenges facing TEI flux measurements are linked to process studies that collect sediment cores, pore waters, sinking material or seawater in close contact with sediments. However, such sampling has not routinely been viable on GEOTRACES expeditions. In this article, we recommend approaches to address these issues: firstly, with an interrogation of emergent data using isotopic mass-balance and inverse modelling techniques; and secondly, by innovating pursuits of direct TEI flux measurements. We exemplify the value of GEOTRACES data with a new inverse model estimate of benthic Al flux in the North Atlantic Ocean. Furthermore, we review viable flux measurement techniques tailored to the sediment–water boundary. We propose that such activities are aimed at regions that intersect the GEOTRACES Science Plan on the basis of seven criteria that may influence TEI fluxes: sediment provenance, composition, organic carbon supply, redox conditions, sedimentation rate, bathymetry and the benthic nepheloid inventory.

This article is part of the themed issue ‘Biological and climatic impacts of ocean trace element chemistry’.

## 1. Introduction

Seawater chemistry controls the conditions for life in the ocean, and, by influencing ecosystem structures and biological productivity, it both forces and responds to ecological and climatic changes [1]. Trace elements and their isotopes (TEIs) play important roles as ingredients and tracers of these fundamental ocean processes, and their occurrence results from exchanges between the solid Earth and ocean waters. An explicit aim of the GEOTRACES Science Plan is to measure the sources and sinks of TEIs in the oceans, so that we can understand TEI cycles and accurately predict their response to, and impact on, global change [2]. At a workshop meeting of The Royal Society, held at Chicheley Hall in December 2015, we considered current knowledge of the mechanisms and rates of TEI exchanges at the four ocean boundaries: the atmosphere, mid-ocean ridges, continents and marine sediments. This article discusses the state of knowledge concerning TEI fluxes at the last of these, the sediment–water boundary. We consider our ability to parametrize TEI fluxes from this boundary in global ocean biogeochemical models, and assess the viable tools to make such necessary measurements. We propose a set of critical factors that need to be considered in flux determinations and make recommendations for future research strategies.

### (a) Sediments as a source and sink for trace elements and their isotopes

**Figure 1.**
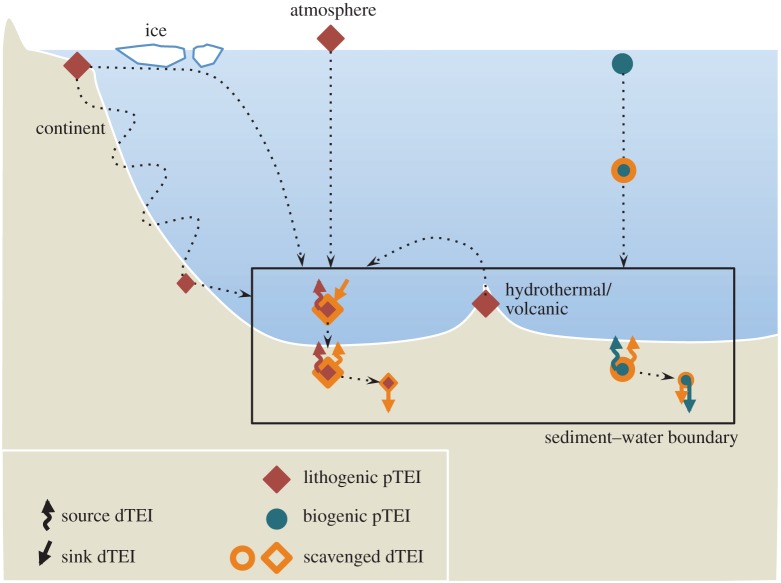
Source and sink pathways of dissolved trace elements and isotopes (dTEI) at the ocean's sediment–water boundary. TEIs enter the sediment–water boundary as lithogenic and biogenic particulates (pTEI) and scavenged dTEIs, where they undergo dissolution and/or burial. Recycling and transport of particulate and dissolved TEIs may occur many times within the sediment–water boundary, between coastal shelves and ocean basins. Fluxes into and out of this zone will mediate the TEI budget of the entire ocean.

The sediment–water boundary is one of four ocean interfaces identified for the exchange of TEIs [2]. Perhaps uniquely, however, ocean sediments are related to properties of the other three interfaces (the atmosphere, continental run-off and the oceanic crust) because these other interfaces all contribute lithogenic material to ocean sediments. To fully characterize the oceanic cycling of TEIs, it is necessary to assess their source–sink properties at the sediment–water boundary (as summarized in figure 1). Lithogenic particulate solids that enter the ocean and settle to the seafloor without ever going through the ‘dissolved’ phase (defined herein as TEIs < 0.2 µm) act as neither a sink nor a source to the chemistry of ocean water. However, if such lithogenic material dissolves on the seafloor, then the products may become a source to the water column. Additionally, if lithogenic or authigenic mineral particulates scavenge dissolved TEIs from the water (or at the seafloor), they become a sink. Biological solids are assimilated from dissolved constituents in seawater, so are a straightforward sink, but if this sink partially re-dissolves at the sediment–water boundary, it becomes a TEI source, serving to offset the extent of its initial removal from the ocean. Building predictive power into ocean biogeochemical and climatic models ultimately depends on our ability to distinguish and quantify the rates of these sources and sinks, but measuring them is not straightforward. Our approaches to measure TEI fluxes routinely integrate the rates of multiple processes, such as the re-dissolution of TEIs after scavenging/bio-assimilation in addition to TEI dissolution from lithogenic material. Measurements of TEI fluxes at the sediment–water boundary are also extraordinarily scarce in comparison with the mapped distribution of TEIs emerging in the era of GEOTRACES, with insufficient knowledge to predict their variability in time and space. This limits our ability to simulate the distribution of TEIs in ocean biogeochemical models and thus predict their response to, and impact on, global change.

### (b) Data constraints for benthic fluxes of trace elements and their isotopes in models

Desirable properties for any flux parametrization in a model are a data-constrained magnitude, and knowledge of the properties that control it. The benthic flux of many TEIs has previously been evaluated, for instance, using the balance of sediment rain and sediment burial rates in ocean basins. By difference, these terms estimate the oceanic inputs of many bio-essential (Fe, Mn, Ni, Cu, Co, Cd and Zn) and tracer (U, Th and Pb) elements for a handful of abyssal sites in the equatorial and mid-latitudinal Pacific and Atlantic Oceans, where sediment pore waters have also been used to evaluate the fluxes of Cu, Ni and Mn. This earlier work is collated in excellent detail by previous reviews (Chester [3], chs. 12 and 13), and we discuss the principal strengths and limitations to the methods used in §§2a and 2b. Importantly, though, these data suffer from limited coverage and only coarse resolution of source and sink terms needed to simulate rates in ocean biogeochemical models. We do not know the extent to which these sources and sinks reflect ‘new’ and/or regenerated sources of TEIs, what criteria are controlling the rates observed, and how the criteria are distributed in time and space.

**Figure 2.**
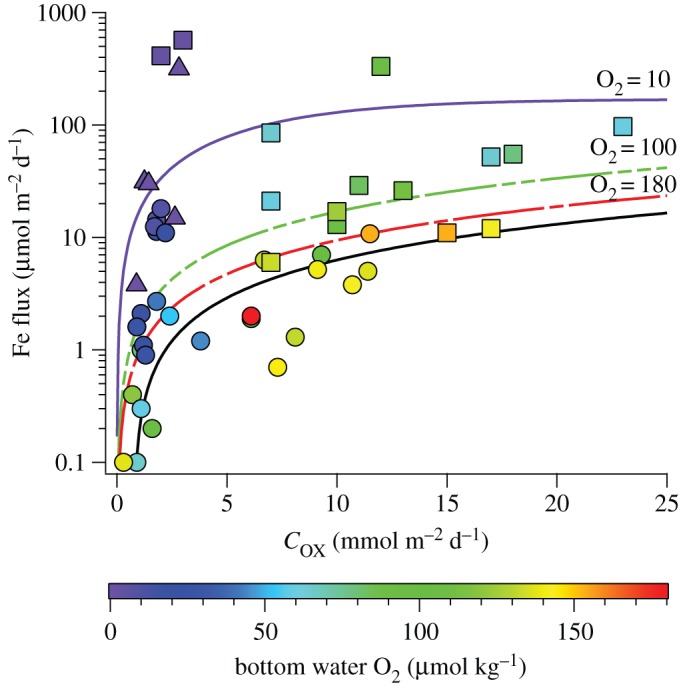
Benthic Fe flux measurements and parametrizations. Data markers correspond to *in situ* measurements as a function of organic C oxidation rates (*C*_OX_) and bottom water oxygen concentrations from Pacific Ocean margin sites after Elrod *et al*. [5] (circles), Severmann *et al*. [7] (squares) and Noffke *et al*. [8] (triangles). The solid black line is the Fe flux parametrization first described by Elrod *et al*. [5]. Dashed lines correspond to Fe flux parametrizations described by Dale *et al*. [6], which account for changes to *C*_OX_ and bottom water oxygen values in all data.

**Figure 3.**
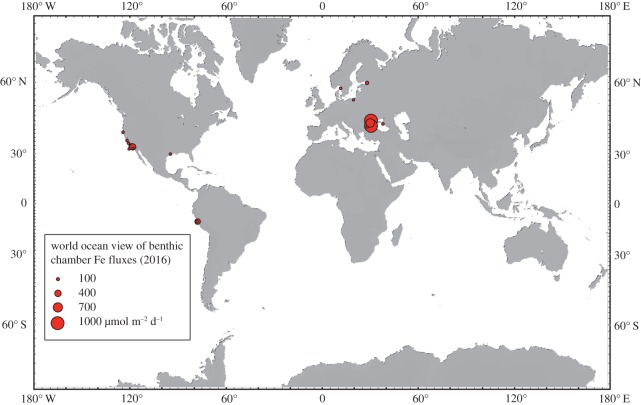
World ocean distribution and magnitude of benthic Fe fluxes derived by *in situ* benthic incubation chambers. Data presented correspond to minimum flux estimates (where available) determined from individual study sites. The compiled data reflect more than 10 individual research studies between 1989 and 2012 [5,7–15], covering five different ocean regions, but note that only two of those regions (California/Oregon margin and Peru margin) are straddling the open ocean. To the best of our knowledge, very few (if any) comparable data exist for the benthic fluxes of other oceanic trace elements. This map was generated using GeoMapApp (http://www.geomapapp.org) [16].

Our understanding of some benthic TEI fluxes is built from more extensive field observations using multiple methodologies. Iron has undergone perhaps the most rigorous investigation of its fluxes over the past two decades, since the recognition of its widespread limiting or co-limiting effect on phytoplankton productivity [4] and the importance of sediment dissolution for the oceanic Fe budget [5]. The result of these field observations is an empirical basis for prediction of the benthic flux of Fe to the ocean that relies upon the observed correlation of Fe flux with organic C oxidation rates at the seafloor [5]:
1.1
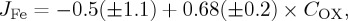

where a benthic Fe flux (*J*_Fe_) in units of µmol m^−2^ d^−1^ is a function of the organic C oxidation rate (*C*_OX_) in units of mmol m^−2^ d^−1^. This regression is underpinned by mechanistic knowledge of these coupled dissolution processes during early sediment diagenesis. A recent refinement now adds bottom water oxygen concentration (O_2BW_) as a second master variable to this equation, which principally governs the efficiency of authigenic Fe oxide entrapment that suppresses dissolved fluxes of reduced Fe species, such that a predicted benthic Fe flux (*J*_Fe_) becomes [6]:
1.2
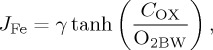

where *γ* is a maximum steady-state flux set at 170 µmol m^−2^ d^−1^ (figure 2). Although these equations for Fe are the best we have to predict sediment fluxes for any TEI, they still suffer from limitations because the data constraints stem from a single method of flux measurement and from very few regions of the ocean (figure 3). As such, these equations inherit all methodological or sample location biases intrinsic to the individual flux measurements. This impacts the validity of ocean biogeochemical models to a degree that is potentially significant but presently uncertain.

**Figure 4.**
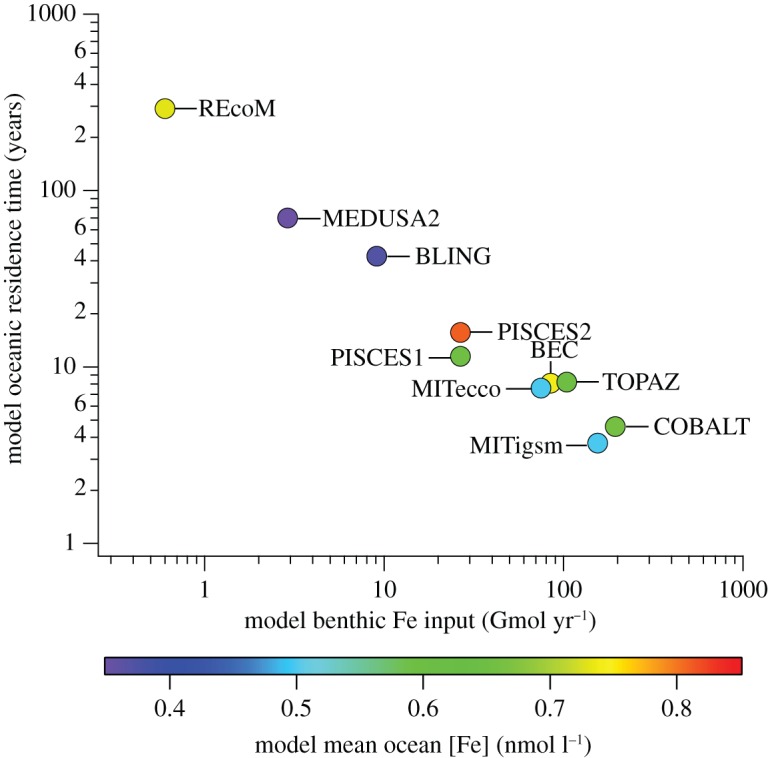
Oceanic residence times of Fe as a function of benthic Fe flux parametrizations in 10 global ocean biogeochemical models, after Tagliabue *et al.* [20]. The colour scale describes the resultant mean seawater concentration of Fe in each model. The range in mean Fe concentration across all models is maintained within a relatively narrow range (0.35–0.83 nmol l^−1^) by adjustment of the scavenging efficiency of Fe to accommodate for variations in benthic Fe flux parametrization.

Measurements used to derive equations (1.1) and (1.2) were made by *in situ* incubation chambers, which have many advantages (see §2a), but cannot account for TEI scavenging in benthic boundary layers (BBLs), or dissolution promoted by sediment resuspension. Microcosm experiments have demonstrated that such processes can be highly effective in modulating benthic Fe fluxes [17], and GEOTRACES section data confirm their widespread importance [18], where diverse particulate mineralogy also has a dramatic influence on adsorption properties [19]. A recent intercomparison of 13 global ocean biogeochemical models has identified uncertainty in the benthic flux of Fe [20] as a major source of model difference. Benthic fluxes of dissolved Fe vary greatly between models. Of the 10 models that include a benthic source of Fe, scavenging of Fe and modification of its oceanic residence time are used to optimize model fits to the observed oceanic distributions of Fe to accommodate for differences in the prescribed benthic Fe flux across three orders of magnitude (figure 4). The measured global benthic Fe flux that is scaled up from *in situ* incubation chambers (approx. 100 Gmol yr^−1^ [5,6]) is at the upper end of the range employed by these models, and requires a very short oceanic residence time for Fe (approx. 10 years), or else the measured fluxes must be substantially overestimating the amount of dissolved Fe truly escaping the sediment.

The equations for benthic Fe flux (equations (1.1) and (1.2)) do usefully add predictive capability to models that also simulate changes to organic C and oxygen in the oceans. However, sediment dissolution processes that occur independently of these master variables remain unaccounted for. The non-reductive dissolution (NRD) of lithogenic material is known to be important, but is presently missing for the oceanic budgets of many elements, including Fe [21]. There are diverse lines of Fe isotopic evidence from Pacific, Atlantic and Southern Ocean regions to indicate that a substantial fraction of dissolved Fe in the ocean may be supplied by NRD processes (see §§2a and 2f). The factors controlling NRD rates are uncertain, but probably reflect the solubility of the silicate minerals present [22–24], physico-chemical conditions (i.e. surface area/grain size and the energetics of physical denudation), as well as TEI cycling during authigenesis [25,26]. Although the rates and occurrences of such dissolution processes will vary geographically, understanding them is vital if we are to accurately predict variability in the oceanic cycling of TEIs.

Benthic Fe fluxes are, therefore, only partially accounted for by present model parametrizations. The impact of solute–particle interactions in the BBLs and of NRD processes are most critically unaccounted for, and have the potential to radically alter the distribution and magnitude of benthic Fe fluxes, and their perceived role in ocean biogeochemical cycles and climate. Other TEIs of major interest, including other bio-essential trace elements (e.g. Mn, Ni, Cu, Co, Cd and Zn), toxicants (e.g. Pb, As, Hg) and tracers such as the rare earth elements (REEs), have received much less attention. These TEIs lack the spatial coverage of flux measurements and broad mechanistic knowledge needed to match the state of knowledge only touched upon here for Fe. Thus, for many TEIs, our understanding and measurement of benthic flux are almost starting from scratch.

In the following section, we outline approaches that may be suited to future measurements of benthic TEI fluxes, and highlight the strengths and limitations of each technique to assist in planning future research. We describe established and emerging techniques that we consider most tractable in the light of the analytical challenges that accompany most TEI measurements at the present time, and show the benefits of using multiple approaches to fill gaps in our knowledge, such as inverse techniques to identify benthic source and sink processes within ocean transect data. In a final section, we also propose a set of criteria for use in planning future investigations of benthic TEI exchange, and we highlight oceanographic regions where diverse sediment properties offer maximum opportunity to link future rate measurements and process understanding with mapped TEI distributions in the water column.

## 2. Tractable approaches to measure a benthic flux of trace elements and their isotopes

### (a) Sediment pore water profiles

**Figure 5:**
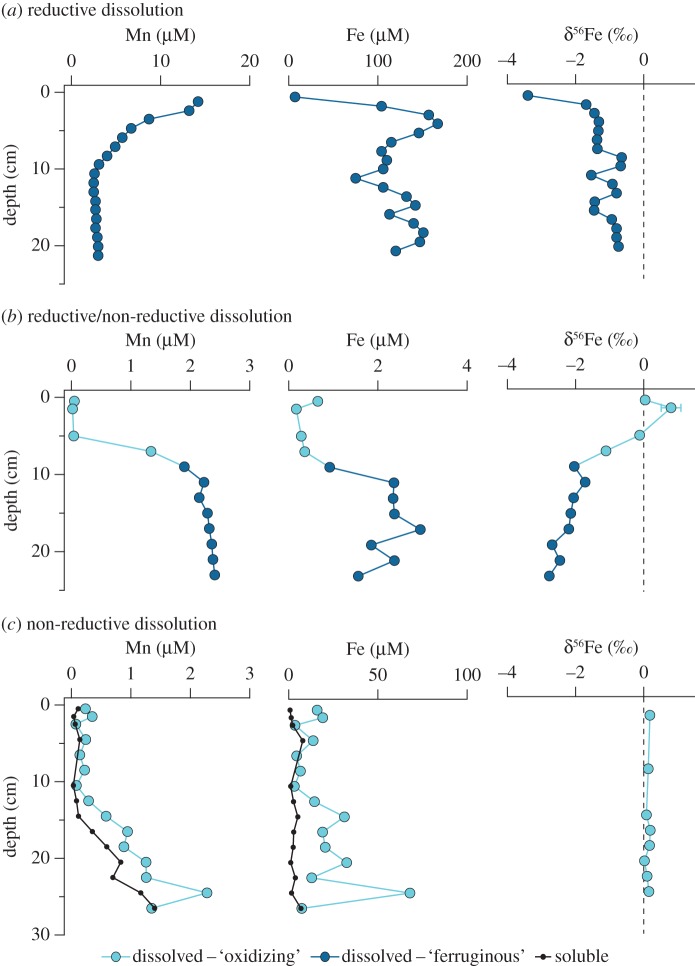
Dissolved (less than 0.2 µm) and soluble (less than 0.02 µm) pore water Fe and Mn profiles in sediments exhibiting reductive and NRD processes, after Homoky *et al*. [26,28,29]. (*a*) Ferruginous pore waters from the Eel River margin, NE Pacific Ocean (110 m), contain substantial pore water enrichments of Fe and Mn in the absence of O_2_ and NO_3_^−^ (not shown), with light δ^56^Fe values consistent with the reductive dissolution of Fe and Mn [28]. (*b*) Oxidizing–ferruginous pore waters from the Cape margin, SE Atlantic Ocean (2662 m), contain minor enrichments of dissolved Fe and Mn, and δ^56^Fe values indicate that a mixture of reductive and NRD processes account for dissolved Fe concentrations [29]. (*c*) Oxidizing pore waters from mixed volcanic/bio-siliceous sediment near the Crozet Islands, Southern Ocean (4222 m), contain large pore water Fe and Mn enrichments despite the presence of O_2_ and NO_3_^−^. Dissolved δ^56^Fe values approximate crustal compositions, and soluble and dissolved Fe and Mn concentrations indicate that NRD of Fe and Mn may promote colloidal species within the ‘dissolved’ pool [28]. Colloidal TEIs will have different properties of diffusion and reaction from their ionic forms, and promote different rates of benthic exchange [26].

Interstitial sediment pore waters may contain the products and reactants of all early diagenetic reactions that sequester, recycle and release dissolved species of TEIs. They are an aqueous continuum of the overlying water column, and in the upper tens of centimetres pore water may comprise anywhere from 50% to more than 90% of the sediment by volume [27]. The relative rates of mineral reaction and solute diffusion produce measurable gradients in dissolved constituents that can be used to quantify the rates of TEI uptake and dissolution (figure 5). Using various transport formulations, pore water flux calculations have provided uniquely powerful (first-order) quantitative constraints of some TEI fluxes to and from seawater, e.g. in deep pelagic [30,31], volcanic [26], shallow coastal [17,32,33] and glaciated sediments [34]).

In their simplest form, TEI fluxes may be estimated on the basis of a concentration gradient following Fick's first law of diffusion [35]:
2.1
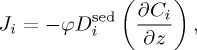

where the flux (*J_*i*_*) of an element (*i*) is the product of its change in concentration (∂*C_*i*_*) over a range in depth (∂*z*) and its effective diffusion coefficient 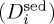 within sediments of specified porosity (*φ*). Effective diffusion coefficients are calculated for specific sediments on the basis of their porosity and tortuosity. Thus, empirically derived diffusion coefficients in seawater 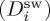 of known ionic strength and temperature are related:
2.2
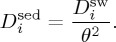

Here, tortuosity (*θ*) is either measured or else well approximated from porosity (*φ*) after Boudreau [36]:
2.3
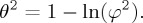

Accuracy of flux calculations firstly depends on the assumption that diffusion is the only mechanism of solute transport, for which accurate assessments of concentration–depth gradient and coefficients of diffusion are then needed. The upper boundary concentration used to derive ∂*C_*i*_* is typically assumed to equal that determined from a Niskin bottle mounted to a multi-corer, or placed within tens or even hundreds of metres above the seafloor by routine shipboard water column sampling methods—any closer than this risks damaging conductivity, temperature and depth (CTD) sensors and water sampling equipment on the seabed. Pore water concentration–depth gradients can be resolved at 0.5 to greater than 1 cm intervals by conventional slicing-centrifuge or squeezing and Rhizon sampling techniques [37,38]. Such pore water extraction methods may yield reasonable sample volumes (e.g. 1–10 ml) to support multiple TEI analyses. A centimetre-scale depth profile may also be appropriate for accurately describing many TEI distributions, but in instances where concentration gradients are very steep across shallow depths beneath the sediment–water interface, gel probe techniques may offer some advantages. Diffusive equilibrium/gradients in thin-film (DET and DGT) gel probes can enhance the depth resolution of pore water samples and even offer two-dimensional mapping of pore water solutes down to submillimetre scales for the calculation of fluxes [39,40]. However, DET and DGT methods yield comparatively small samples, which will be more challenging for many low-abundance TEI measurements in open ocean settings. Further, any approach to pore water sampling should ensure artefacts of TEI distribution are minimized (e.g. [41]). Diffusion coefficients of free ions in seawaters have been empirically derived, but uncertainty arises when we assume the ‘dissolved’ (less than 0.2 µm) measure of TEIs represents purely ionic species in pore waters. Assessments of soluble and colloidal TEIs in pore waters are largely missing, yet colloids are shown to be a significant and variable fraction of dissolved Fe and Mn in marine sediments (figure 5*c*) [26]. Thus, ionic diffusion coefficients may not always be suitable transport descriptors of TEIs.

There are other approaches used to calculate solute fluxes during early diagenesis that go beyond simple diffusion. Perhaps most famous are the diagenetic equations of Berner [42,43], which combine the ionic diffusion described by Li & Gregory [35] with the processes of organic matter decomposition, mineral dissolution and precipitation, adsorption and ion exchange, bioturbation, compaction-driven advection and burial. Berner in 1976 described these flux calculations as ‘often being fraught with difficulties due to: incorrect formulation and estimation of gradients and of diffusion coefficients; lack of an evaluation of the role of turbulent mixing at the sediment–water interface due to waves, currents, and bioturbation; lack of correction for depositional burial of pore waters; and lack of consideration of diffusion within the viscous sub-layer of bottom water’ [44]. These difficulties are just as prevalent today, and are most strongly expressed in shallow water environments. Berner suggested that, where deposition and bioturbation are major variables, preference should be given to *in situ* flux measurements (§2b). Indeed, while few published intercomparisons of these approaches to benthic flux measurement exist, pore water and *in situ* chambers in such settings might only agree to within an order of magnitude [9,17,45,46]. Agreements between fluxes derived by benthic chambers and those from pore water profiles are especially poor in environments with significant biophysical disturbances [5,7,32,47]. By contrast, in deep-water pelagic environments, where these influences are less pronounced, it might be preferable to use simple Fick's first law calculations (equation (2.1)) for a reasonable estimate of fluxes [26]. The same might also be true for laminated sediments beneath an oxygen-deficient water column where bioturbation and bioirrigation are suppressed [8].

One vertical dimension and a steady state are typically assumed in pore water flux calculations, and regional, basin-scale and global budgets must be extrapolated from the small-scale observations attributed to sediment coring campaigns. Such flux derivations routinely neglect the variations in time and space of important flux-modulating factors, such as multiple sources of advection, or scavenging rates in bottom waters. For example, advection may dominate solute fluxes: (i) due to bottom currents and topography over coarse permeable sediments [48,49], (ii) via animal activity [32,40,47] and (iii) by diffuse hydrothermal promotion of vertical solute transport in sediments over ridge axis and flank environments [50]. Despite this diversely driven yet common property, we struggle to capture the whole influence of advection (e.g. [49]) in even the most sophisticated benthic flux determinations for TEIs (e.g. [5,6], §2b). Further, physico-chemical transformations in the BBLs have additional moderating effects on the release, scavenging and exchange of TEIs [17,18,21,51,52] that pore water fluxes cannot account for by themselves.

Understanding what controls the distribution of TEIs in pore water is equally important to quantifying fluxes, and essential if we are to build predictive power into ocean biogeochemical models. Sediments and pore waters may usefully contain such valuable information for the study of TEI exchange processes. For example, organic carbon burial is a major driver of early diagenetic reactions and benthic exchange [5,30,53–56], but properties inherent to the mineral substrates and their proportional abundance will also influence their mechanisms and subsequent rates of dissolution. Volcanic minerals, for example, provide substantial inventories of dissolved Fe and Mn in marine sediment pore waters in the absence of appreciable organic matter [26,50], and the isotopic composition of this Fe indicates that NRD (or oxidative weathering) of lithogenic material may be responsible for the large inventories of dissolved Fe and Mn observed [28]. Even in more refractory continental margin sediments, Fe isotopes indicate that oxidative weathering may sustain a small fraction of ‘dissolved’ iron in pore waters without the chemical reduction and isotopic fractionation of Fe attributed to organic matter oxidation [29] (figure 5). In this respect, sediments and pore waters provide vital clues that help formulate our approach to quantifying fluxes (§2f), and there is scope to deepen our knowledge of solid–colloidal–aqueous transformations for TEIs through complementary experiments.

A challenge for all pore water studies is sampling an adequate spatial resolution beneath the surface boundary, where data quality may greatly influence the accuracy of gradients observed. Added to which, even the most precise *ex situ* pore water sampling techniques will reflect decompression artefacts that may adsorb, desorb or precipitate TEIs during sediment recovery to uncertain degrees. Many of the TEI measurements being made in the water column by the GEOTRACES programme have rarely, if at all, been measured in sediment pore waters. Nor have pore water sampling protocols been subject to the rigours of GEOTRACES inter-calibration efforts, which have ensured the accuracy and reproducibility of water column data by the coordinated development of TEI-clean sampling protocols. Of the micronutrient trace elements, for example, pore water data for Fe and Mn are most widely reported, while Cu, Cd and Ni are limited to a handful of studies [30,32,39,53], and pore water Zn data are absent in all but a few exceptional deep ocean drill-core and estuarine examples [39,57–59]. For TEI tracers, such as the REEs, quantifications are also scare, despite robust evidence from pore waters and GEOTRACES section data indicating that sediment sources are responsible for determining seawater REE patterns and Nd isotopic compositions [33,60–63]. The present lack of detailed pore water data from diverse sedimentary environments means we are lacking some of the most basic knowledge required to understand and calculate fluxes of these important TEIs between sediments and the ocean.

#### (i) Summarized utility of sediment pore water profiles

*Advantages*

— Pore waters provide a relatively simple method to derive diffusive TEI fluxes.
— Pore waters may reveal mechanisms controlling TEI fluxes.
— Sediment coring is a relatively efficient use of ship time that can complement water column work provided that the ship has winch capabilities and controlled temperature laboratories.
— Sediment coring supports ancillary TEI flux methodologies (e.g. §§2c and 2d).
— Surface sediments and pore waters connect observations of modern TEI cycles to geological archives.

*Limitations*

— Pore water sampling is sensitive to *ex situ* redox, temperature and pressure changes.
— Adaptions for larger pore water volumes and/or cleanliness (e.g. Zn) may be required for new TEI measurements.
— Depth profiles can be impacted by local and small-scale heterogeneity of the seafloor.
— Steady-state pore water assumptions may be invalid during dynamic macro faunal activity, or seasonal and stochastic sedimentation events (e.g. phytodetrital and turbidite inputs).
— Knowledge and/or assumptions of TEI speciation and diffusion coefficients are not always representative of the ‘dissolved’ pool of TEIs.
— Pore waters do not capture the influence of bottom-driven advection and/or scavenging within nepheloid layers.

### (b) *In situ* benthic incubation

*In situ* benthic chambers are designed to trap seawater in contact with the seafloor and sample the chemical properties of seawater over time. Such incubation experiments can provide a direct measure of net elemental exchange. These techniques have evolved from simple bell jars upturned by scuba divers in shallow water sediment settings [32] to self-powered ‘free-vehicle’ benthic landers with multiple sample chambers, ancillary data logging and mechanical sample extraction and storage, suitable for deployments in deep waters [64,65]. There are other types of *in situ* landers also designed to measure chemical fluxes. For example, three-dimensional eddy-correlation techniques derive a vertical flux by measuring two parameters simultaneously and at a fixed point above the sediment–water interface over time: the fluctuating vertical velocity and a fluctuating chemical property, like O_2_, can be used to calculate the vertical flux of O_2_ [66]. This non-invasive technique has the advantage that it can fully account for diffusive and advective transport mechanisms. Further, boundary conditions such as bottom water O_2_ are not artificially altered over the course of the measurement, as is the case in benthic chambers [7,10,11]. While eddy correlation is an elegant approach, it demands fast-response, low-consumption, *in situ* chemical microsensors, which are not presently available for the majority of TEIs that are of interest in the ocean, and so herein we focus on the more tractable results offered specifically by *in situ* benthic incubations.

**Figure 6.**
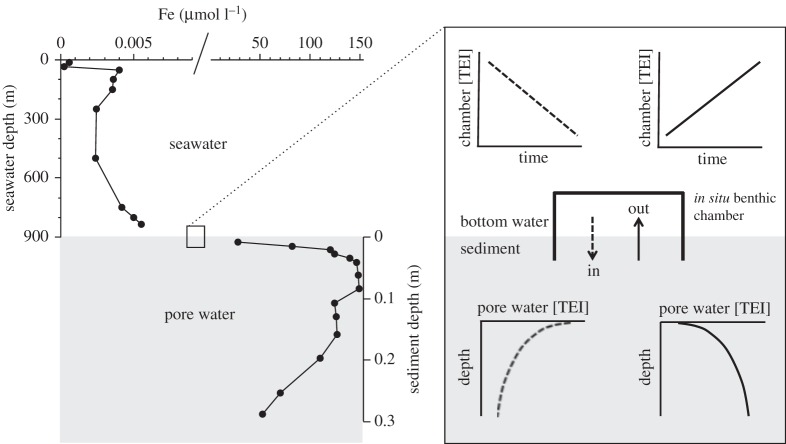
Dissolved Fe in the San Pedro Basin, California, and *in situ* approaches to measures TEI exchange at the sediment–water boundary. Pore water data are reproduced from McManus *et al*. [67] and water column data from John *et al*. [68]. Note that dissolved Fe concentrations undergo fourth-order spatial-scale and concentration changes at the difficult-to-sample sediment–water boundary, where rates of exchange need to be measured. Uniquely, *in situ* incubation chambers use temporal rather than spatial gradients in TEI concentrations to evaluate rates of exchange.

Common to all *in situ* benthic incubations is the principal measure of TEI exchange, which is summarized schematically in figure 6. This method relies on capturing a water sample at discrete time intervals for subsequent shipboard or laboratory analyses. In theory, the sampling approach is suited to many of the analytical methods already developed for numerous TEIs in seawater, as long as adequate cleanliness of the instrument can be assured and the necessary sample volumes obtained. An element flux (*J_*i*_*) can be calculated based on a change in element (*i*) concentration with time (figure 6), such that
2.4
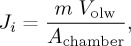

where *m* is equal to the slope of the regression (or 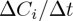), *V*_olw_ is the volume of overlying water trapped within the chamber and *A*_chamber_ is the surface area of sediment within the chamber.

*In situ* benthic incubations have been pivotal in the quantification of organic carbon recycling in the ocean [69–71] and the regeneration of important macronutrients, micronutrients [5,7,8,11,45,46,67,71–73] and their isotopes [7]. Such incubations benefit from directly measuring the net exchange that results from the transport and reaction of many ‘dissolved’ species, including any ionic, ligand-bound or nanoparticulate forms present in the ‘dissolved’ pool, and the influence of bioirrigating animals. By capturing a larger surface area of the seafloor than a typical sediment core, e.g. [12], benthic chambers also help to integrate the patchy influence of discrete animal burrows.

Fluxes have been observed for a number of TEIs, including U, Fe, Mn, Ba, Cu and Rn, in addition to key macronutrients (O_2_, silicic acid, phosphate, nitrate, ammonia and carbonate). By comparing TEI measurements with other properties, such as oxygen, it has been possible to find empirical relationships that are used to predict spatio-temporal changes to benthic TEI fluxes. In the case of Fe, and on the basis of relationships with organic C oxidation rates [5] and bottom water oxygen concentrations [6,7], this approach has produced a most recent estimate of benthic iron fluxes from the global ocean floor [6], 109 ± 55 Gmol yr^−1^ (0–2000 m) [25,33], of which 37 Gmol yr^−1^ is from slope sediments (200–2000 m), with an additional 44 ± 21 Gmol yr^−1^ predicted from sediments beneath 2000 m. Thus, these *in situ* chamber data have also empowered model experiments of ocean biogeochemical cycles, which can use these real-world rate estimates to study ocean-coupled climate variability [74,75] (see §1b).

Present global coverage of *in situ* benthic incubation studies, however, is remarkably sparse. In the case of Fe, the empirical relationships described first by Elrod *et al*. [5], Severmann *et al*. [7] and then Dale *et al*. [6] were predominantly derived from coastal sites in two regions of the eastern Pacific Ocean, with comparatively low bottom water oxygen, high organic matter and terrigenous supply. Although these sites do cover a reasonable range of benthic conditions, such as the supplies of lithogenic solids, reactive iron minerals and organic C, beneath a range of bottom water O_2_ concentrations, their magnitudes and depth distributions are unlikely to be representative of global ocean margins [29]. Other estimates from some enclosed basins are also available, but they have only limited application to the open ocean [11]. The study of other sediment types (e.g. volcanic, low carbon) and new oceanographic provinces (e.g. oxic, slow sedimentation rate) that potentially reflect more prominent roles for other sediment dissolution mechanisms [29,76] is now needed, and motivates our recommended regions for future investigation (§3a), where the application of *in situ* benthic incubations to many additional TEIs also has unexplored potential.

The challenges facing the application of benthic chamber methods to a broader range of TEI fluxes concern its sensitivity to TEI rates of exchange. For example, during 1- to 2-day incubations on continental shelves, the release of Fe and depletion of O_2_ inside the chamber can be substantial, such that fluxes of reduced Fe must be derived from the initial and more conservative concentration changes (e.g. 0–4 h), before the oxidation kinetics of Fe are excessively perturbed and produce artefacts in elevated Fe concentration in the chambers [7,10,11]. In the case of dissolved Mn, for which the oxidation kinetics are slower, the incubation duration may be optimum to observe fluxes [72], although it too has been shown to be subject to artefacts during longer chamber deployments. For TEIs with much slower rates of benthic exchange, a much longer period of incubation might be needed to resolve the concentration changes, during which time the inherent risk of perturbing the natural state of variables like oxygen, pH and animal activity becomes even greater, potentially even prohibitive. Such problems become even more difficult to overcome for conservative elements with relatively high background concentrations, such as U, Mo, Re, V and Ca, and therefore need careful consideration. Additionally, and inherent to all TEI flux measurements by benthic incubations, the role of bottom currents in driving pore water advection [49] and suspended particle–solute interactions are not well represented [17]. This conclusion is supported by Dale *et al*. [6], whose reevaluation of *in situ* benthic Fe flux data predicts a more prominent role for particle scavenging of dissolved iron in the global ocean (see also §1b). *Ex situ* incubation experiments have been used successfully in concert with *in situ* incubations to study the effects of boundary layer particle suspensions on benthic Fe fluxes [17], but replicating these conditions to derive accurate rates *ex situ* can pose even greater challenges [12,17], so combining *in situ* and *ex situ* approaches, or adding experimental controls to physical properties *in situ*, is recommended for future campaigns.

#### (i) Summarized utility of *in situ* benthic incubation

*Advantages*

— Proved ability to measure benthic fluxes (release or uptake) of some TEIs.
— Captures the influence of burrowing/irrigating animals in sediments.
— Measures exchange rate without need for knowledge of TEI speciation or coefficients of diffusion.
— Improved spatial coverage of seafloor compared with pore water profiles.

*Limitations*

— Presently suffer from low coverage of global seafloor environments.
— Incubation times may not be suitable for some TEI rates of exchange.
— Do not capture the influence of bottom-driven advection and/or scavenging within nepheloid layers.
— More challenging deployment and recovery of free-vehicle benthic landers during research expeditions.

### (c) Moored sediment traps

Sediments traps provide an elegant means to evaluate TEI sources and sinks in the oceans in combination with TEI burial rates recorded in sediments. However, the measured terms used to calculate a flux reflect drastically mismatched time scales. By capturing the particulate rain of TEIs sinking through the water column over time and comparing these values to TEI burial rates in seafloor sediments, a measure of dissolved benthic TEI flux can calculated:
2.5
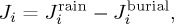

where a benthic flux for a given element (*J_*i*_*) is equal to its particulate supply through the water column 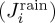 minus its rate of burial (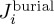). This relationship can be used to describe both dissolved-source and scavenged-sink terms for TEIs in instances where rain rates exceed burial rates or vice versa. The approach has been used to derive benchmark determinations of TEI fluxes in both the Atlantic and Pacific Oceans, and is also a method that helped to pioneer our understanding of pelagic remineralization rates for major oceanic provinces (see [3] for a more thorough review).

Sediment traps come in many forms, and may be deployed across various time scales from days to months to years, with or without the ability to capture time-series (e.g. seasonal) particulate samples. There are some important criteria for their deployment. Firstly, it is necessary to sample a range of depths throughout the water column to distinguish the primary flux of sinking pelagic and lithogenic material from the resuspended flux of sediment entrained from near-bottom nepheloid layers. Gardner *et al*. [77] describes an effective three-layer approach to ocean sampling with sediment traps. Secondly, an adequate duration of sampling must be planned to enable reasonable intercomparisons with sediment burial rate measurements. In practice, the duration of sediment-trap deployments is routinely compromised for calculations of *J_*i*_*, because burial rates (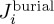) are integrated over much longer time scales in sediment cores (10^1^–10^3^ years) than would be feasible for the measurement of sinking particles by sediment traps (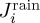). As such, trap deployments over a full seasonal cycle ought to be the pragmatic bare minimum to support dissolved TEI source and sink evaluations. Uncertainty will be minimized for benthic TEI exchanges that represent a large fraction of their particulate rain rates. The propagation of error between these independently measured values, however, is likely to prohibit the sensitivity of the method for small TEI exchanges over the time scales represented. Sediment traps may also prove to be poorly suited for many marginal settings, where dynamic BBLs and significant downslope sediment transport are commonplace. By contrast, moored sediment traps are most suited to open ocean and deep-water settings, where spatio-temporal variation to particulate rain is at a minimum in the ocean, and sediment accumulation is dominated by vertical fluxes.

#### (i) Summarized utility of moored sediment traps

*Advantages*

— Sediment traps provide temporally and spatially averaged measures of sinking particulate TEI compositions and rain rates in the ocean.
— Measurements of TEIs in particulate and sediment samples may offer more generous inventories for analytical methods than seawater or pore water samples.
— Composition of buried and sinking particulate material might be used to distinguish lithogenic and biogenic TEI sources.
— The effect of suspended TEI scavenging/dissolution at the sediment–water boundary is accounted for.

*Limitations*

— Calibration of trapping efficiency is essential to improve flux accuracy.
— Repeat expeditions are needed to support long-term deployment and recovery of sediment traps.
— Sediment traps are poorly suited for measuring fluxes in marginal ocean environments, and in areas of dynamic sediment transport.
— Sediment coring is an essential counterpart for the determination of TEI burial rates.
— Sediment with continuous laminar sedimentation is necessary to constrain accurate burial rates to calculate TEI exchanges.
— Burial rates will reflect long-term averages (likely to be thousands of years) compared with annual integrations for sinking particle flux.

### (d) Sedimentary records of benthic trace element exchange

Sediments are the ultimate records of benthic TEI sources and sinks. While the water column records TEI variations on daily, seasonal or decadal time scales, sediments provide an integrated record of TEI exchanges over time scales of hundreds to thousands of years. The benthic depletion (source) and enrichment (sink) of TEIs exchanged with the ocean is recorded in the bulk sediment composition as a deficit or excess of TEIs relative to the ‘lithogenic baseline’ [78]. This baseline represents the bulk weathered but otherwise unaltered rock as it enters the ocean, and stands in contrast with authigenic or biogenic minerals forming within the ocean. Sediment enrichment or depletion of TEIs can be expressed as simple elemental ratios, where a metal of interest is normalized to a typically abundant and refractory lithogenic element or isotope (e.g. Al, Ti, ^232^Th). In many cases, the lithogenic baseline can be assumed to closely match the composition of average continental crusts, but for some elements this assumption is less robust and local knowledge of baseline composition is needed. An alternative approach is to isolate the enriched fraction either stoichiometrically [78–80], by subtracting the lithogenous fraction of the element, or chemically, by extracting non-lithogenous phases (e.g. [81]). Chemical extraction methods may be advantageous for elements with high lithogenic baselines, or where specific mineral phases are targeted, and there is a large body of literature on extraction methods for sediments (e.g. [81–85]) and suspended particles [86–88], but often little consensus on the optimum technique. Trace element enrichments provide powerful records of many TEI sinks for use in palaeoceanography, because many metals are especially sensitive to bottom water redox and palaeoproductivity variations [79]. In all cases, however, the rates of TEI enrichment or depletion in the sediment record rely on knowledge of sediment accumulation rates (e.g. [89–91]), for which the accuracy of dating techniques will largely dictate the accuracy of TEI flux calculations. To explore one such an example in more detail, here we consider the use of ^230^Th, a constant-flux proxy that can provide a quantitative assessment of the sink of some TEIs to sediments. Also, with novel approaches, ^230^Th can potentially be used to assess TEI sources from dissolution at the seafloor.

Thorium-230 is generated by radioactive decay of ^234^U dissolved. Uranium is soluble in seawater, and the concentration of ^234^U is not thought to change significantly on time scales less than 10^4^ years, so that production of ^230^Th in seawater is constant in space and time (at a rate of 252 dpm m^−3^ yr^−1^) (dpm = disintegrations per minute). Thorium is highly particle-reactive, so, upon production, ^230^Th is rapidly adsorbed to particles that settle to the sediment. This scavenging process gives ^230^Th an average ocean residence time of ≈20 years [89], making it one of the shortest-lived of all elements in seawater. The rapid removal suggests that all ^230^Th formed in a column of seawater falls to the sediment immediately below that column [92]. In practice, there is some capacity for advection of ^230^Th prior to removal, but sediment-trap studies (e.g. [93,94]) and modelling [95] indicate that this advection does not lead to deviation of more than ≈30% from the assumption that all ^230^Th is removed to sediment immediately below its site of formation. This quantitative removal means that the flux of ^230^Th to the seafloor is constant, set only by the depth of overlying seawater.

As the flux of ^230^Th to sediment is known, the flux of other components can be assessed from the mass ratio between that component and ^230^Th in the sediment. This approach relies on the equation [96]
2.6
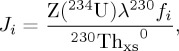

where *J_*i*_* is the Th-normalized flux of a TEI, *i*, to the sediment (g m^−2^ yr^−1^), *f_*i*_* is its weight fraction in the sediment, *Z* is the water depth (metres), (^234^U) is the activity of ^234^U in seawater (= 2750 dpm m^−3^) and *λ*^230^ is the decay constant of ^230^Th (year^−1^). The subscript xs on ^230^Th_xs_^0^ indicates that the concentration (dpm g^−1^) must be corrected for any ^230^Th present in lithogenic material, rather than scavenged from seawater, and the superscript 0 indicates that the concentration must be age-corrected if not modern. These corrections, and the general use of ^230^Th as a constant-flux proxy, are described in more detail in Henderson & Anderson [89].

A prime example of the use of ^230^Th to provide fluxes of a TEI into marine sediments is that of authigenic uranium. Although soluble in oxic seawater, U(vi) is reduced to its insoluble U(iv) state in reducing sediments. As insoluble U moves to the solid phase in such sediments, the resulting decrease in pore water U concentration provides a concentration gradient relative to seawater and a diffusional flux of U into the sediment [97]. This flux represents one of the major sinks in the global ocean cycle for U [98]. It varies in space and time due to supply of organic carbon to sediments and, to some extent, bottom water oxygen, but ^230^Th normalization allows the processes that control this variation to be understood and quantified.

Assessment of the removal of dissolved U to sediment requires authigenic U to be distinguished from U hosted in lithogenic material, which passed through the ocean from continental sources but was never dissolved in seawater. These two sources of U can be separated by measurement of an element whose sedimentary concentration is dominated by the lithogenic fraction, and by assuming a typical continental composition for that material. For authigenic U, ^232^Th is commonly used to assess sedimentary lithogenic content, and authigenic U concentration is then given by
2.7
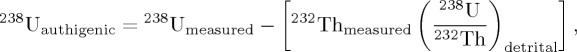

**Figure 7.**
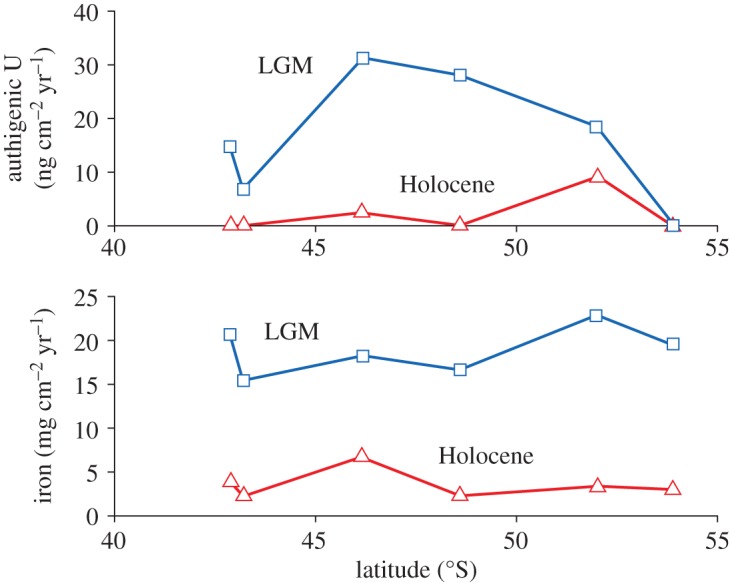
^230^Th-normalized fluxes to the sediment of U and Fe for six cores spanning the frontal systems of the South Atlantic (LGM = Last Glacial Maximum), following Kumar *et al*. [99]. For U, which is significantly enriched in sediment due to authigenic uptake, this flux signifies removal of dissolved U from seawater. For Fe, which does not exhibit this level of authigenic enrichment, the flux is for total Fe, and is likely to be dominated by detrital Fe that has never been dissolved in seawater. (Online version in colour.)

Combining resulting concentrations of authigenic U with ^230^Th normalization (i.e. equation (2.6)) quantifies the removal flux of U from seawater. An example of this approach is the study of a series of sediment cores from 43 to 54° S in the Atlantic by Kumar *et al*. [99]. By combining ^232^Th_xs_^0^ measurements with ^232^Th and ^238^U concentrations, fluxes of authigenic U were reported, ranging from zero to more than 3 ng cm^−2^ yr^−1^ (figure 7). Although that study focused on the use of authigenic U as a tracer of productivity, it also illustrated how ^230^Th normalization provides quantification of TEI fluxes to sediment, and information about the relationship between such fluxes and the local ocean environment.

A similar approach can be used to assess removal fluxes for other elements that undergo authigenic enrichment in marine sediments. Morford & Emerson [100], for instance, assessed concentrations of authigenic V, Mo, Re and Cd (in addition to U) for sediments from the northwest African margin. They used sedimentary Al concentrations, and typical crustal ratios, to assess the detrital content for each metal and derive authigenic concentration. A number of other studies have assessed the role of redox or carbon delivery in influencing TEI concentrations in sediments, with Cd being a particular focus (e.g. [55]) because of its additional use as a palaeoproxy for ocean circulation and nutrient utilization. In some cases, mass accumulation rates have been calculated by combining authigenic TEI concentrations with sedimentation rates derived from ^210^Pb measurements [56]. Such studies have typically not coupled ^230^Th normalization to assessment of authigenic TEI concentrations, however, so that removal fluxes of these metals are not well quantified. Such work could be a productive direction for future assessment of removal fluxes of some TEIs from seawater.

Assessing the flux of dissolved metals into marine sediments from sediment chemistry relies on distinguishing authigenic from lithogenic components in the sediment. This is possible for elements that are strongly enriched by authigenic processes, as discussed above, but even then can be challenging due to the variation in composition of lithogenic material. For authigenic U, for instance, lithogenic ^238^U/^232^Th ratios (expressed as activity ratios) are thought to be 0.6 ± 0.1 for the Atlantic, 0.7 ± 0.1 for the Pacific and 0.4 ± 0.1 south of the Antarctic Polar Front [89]. Similar regional variability exists for TEI/Al ratios [78]. Such spatial variability and uncertainty in lithogenic values must be assessed before authigenic fluxes can be calculated, particularly where such fluxes are small. The assumption of lithogenic detritus with a typical upper continental crust composition is a reasonable first approximation, but variability in this value must be considered for accurate assessment, particularly when and where sedimentation is slow, and ideally a local assessment made of lithogenic composition. Variation in the lithogenic composition prevents the application of the approach described here to removal fluxes of some elements. For instance, in most sediments, fluxes of Fe from the dissolved phase to sediments are too small to readily distinguish from detrital Fe. Thorium-230 normalized records of Fe flux to sediment (e.g. [99,101]; figure 7) do not differentiate between dissolved and detrital fluxes, but provide a record of complete Fe supply to the sediment. A similar situation exists for other elements that are not significantly enriched by authigenic processes in sediments, although improved characterization of the local detrital composition may allow an increased range of element fluxes to be assessed.

Two additional approaches to the application of ^230^Th normalization may allow assessment of sources of TEIs from marine sediments to seawater. In the first, comparison of normalized TEI fluxes in near-bottom sediment traps with those in core-top sediment from the same location would enable calculation of the regeneration of material at the seafloor by differencing the fluxes. Such an approach could also provide local assessment of the TEI composition of material before it becomes incorporated in sediment, helping distinguish water column removal processes from subsequent authigenic fluxes into the sediment. Although comparison of co-located sediment trap and sediment data has occasionally been conducted (§2c), quantifying TEI fluxes between them using ^230^Th normalization has not yet been pursued and could be a useful approach to improve understanding of sedimentary fluxes. A second approach is to compare composition at the sediment–water interface with those at slightly greater depth (i.e. deep in, or just below, the sediment mixed layer). Comparison of authigenic fluxes at two depths in this way can provide an assessment of loss or gain during early diagenesis of marine sediment. This approach has been applied to sediment mineralogy [102] but not yet to TEI fluxes. Both approaches—comparing sediment traps with sediments, and sediments at different depths—rely on a constant magnitude and composition of sedimenting material between the two samples. This may be reasonable in many settings, but care should be taken with variable seasonal fluxes or where sedimentation rates are sufficiently slow that sediments age rapidly below seafloor. These approaches have not yet been pursued in the GEOTRACES programme, but offer potential to increase knowledge of the long-term TEI fluxes into and out of marine sediments.

#### (i) Summarized utility of sedimentary records

*Advantages*

— Combining Th or Al normalization with measurement of TEIs in core-top sediments enables quantitative assessment of the fluxes of authigenic TEIs into marine sediments.
— This approach can be pursued on any core-top sediment, so can be incorporated into any cruise undertaking coring, including GEOTRACES section cruises.
— Solid-phase sediment measurements can be conducted in tandem with pore water measurements (see §2a) to provide complementary information about TEI fluxes.
— Combining measurements of authigenic enrichments in sediment traps and core tops, or core tops and deeper sediments, has potential to quantify bulk fluxes of TEIs from sediment to seawater and has not yet been explored.

*Limitations*

— Variation in the composition of detrital material in marine sediments means that normalization to detrital elements cannot provide information about sinks of dissolved TEIs unless they are significantly enriched in sediments due to authigenic processes.
— This bulk sediment approach assesses the long-term fluxes of TEIs, and cannot provide information about short-term change (e.g. seasonal, inter-annual).
— Approaches using near-bottom sediment traps could not be pursued on section cruises and would need to form part of a process study with longer station occupations.

### (e) Benthic boundary layer and radiotracer profiles

The measurement of TEIs in the water column close to the seafloor may provide an evaluation of TEI exchanges into or out of the BBL, and across spatial scales well suited to link with ocean transect data produced by GEOTRACES. The BBL is a physically distinct region of the ocean overlying the entire sediment–water interface and is an integral component of the sediment–water boundary. Typically tens of metres thick, the BBL is defined by the frictional influence of the seabed itself on the circulating ocean. It can be subdivided into further layers of varying thicknesses: the diffusive boundary layer (sub millimetres), the viscous sublayer (tens of centimetres), the logarithmic layer (one to tens of metres) and, in some cases, the Ekman layer (more than tens of metres). Sampling of the BBL is routinely patchy, if sampled at all, by traditional ship and wire-mounted bottle rosettes, yet this region of the sediment–water boundary is the locus of intense vertical and lateral transport of solutes and particles [103]. Vertical mixing over tens of metres in the BBL may integrate tens of horizontal kilometres of the ocean floor [104] and smooth out small-scale variability in chemical fluxes over its region of influence. Further, it promotes dynamic interactions between solutes and particles that will modulate TEI fluxes across the sediment–water boundary [105].

**Figure 8.**
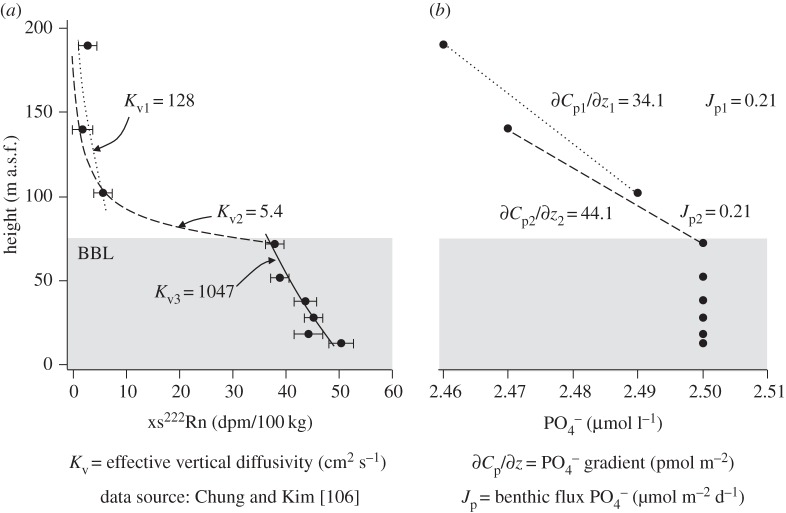
Quantifying chemical exchanges across the ocean's BBL. The distribution of (*a*) the radioisotope ^222^Rn (*t*_1/2_ = 5.6 days) and (*b*) PO_4_^−^ in bottom waters of the Arabian Sea between 4000 and 4175 m below sea level (a.s.f. = above seafloor), adapted from Chung & Kim [106]. Using the authors' derivations of effective vertical eddy diffusivity (*K*_v_), we calculate the effective flux of PO_4_^−^ (*J*_p_ = 0.21 µmol m^2^ d^−1^) using equation (2.6), with a molecular diffusion coefficient for HPO_4_ at 5°C (8.14 × 10^−6^ cm^2^ s^−1^) and the concentration–depth gradients identified by dashed lines. Theoretically, if PO_4_^−^ is supplied only from the sediments and behaves quasi-conservatively in the BBL, a calculation of *J*_p3_ would also be equal to *J*_p1_ and *J*_p2_, despite the very large value of *K*_v3_; however, the corresponding PO_4_^−^ gradient (1.4 pmol/59 m) is below detection of the analytical method.

The transport of solutes in waters above the seafloor is dominated by turbulent eddy diffusivity, and so a quantification of vertical TEI flux across the BBL largely depends on an accurate vertical measurement of this diffusivity (*K*_v_), illustrated in figure 8*a*. Radon-222 (^222^Rn, *t*_1/2_ = 3.8 days) is one of several short-lived radioisotopes that are well suited to quantifying rates of vertical mixing at the seafloor [107]. Common to all such radioisotopes is that they are inert, generated predominantly in the sediments and highly mobile compared with their radiogenic parent. Vertical profiles of these tracers can be used to quantify dispersion of solutes in the bottom water. However, one-dimensional mixing models have proved to yield unreasonable values for *K*_v_ when they are assumed to represent purely diapycnal mixing. Consequently, more sophisticated two- and three-dimensional models are required to capture the complexity of diapycnal and isopycnal mixing processes that control the vertical transport and distribution of solutes in the BBL [108]. However, Sarmiento & Rooth [108] concluded that the influence of isopycnal mixing does not invalidate the use of estimates from radiotracer profiles of the effective vertical eddy diffusivity (*K*_v_) for the purposes of boundary layer chemistry, provided congruence between the tracer source and sources for other elements may be safely assumed.

With an independent measure of *K*_v_, the effective vertical flux of an element (*i*) can be calculated following Boudreau [105]:
2.8
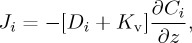

where *J_*i*_* is the flux of *i*, that has a molecular diffusion coefficient *D_*i*_* in seawater, *K*_v_ is the effective vertical eddy diffusivity for a given range in depth (∂*z*) and ∂*C_*i*_*/∂*z* is the concentration gradient of *i* across this depth range. Common to all flux calculations, the reactivity of TEIs can and must also be considered; such issues are discussed in detail by Boudreau [105]. Briefly, in cases where the reaction time scale of a TEI is short, relative to the time scales of transport in the BBL, this would be an important criterion. For example, the oxidation half-life of soluble Fe^2+^ in well-oxygenated bottom waters is of the order of a few minutes, and so precipitation reactions are certain to occur within the time scales for vertical mixing across a BBL, which are of the order of hours to days, in addition to any scavenging or colloidal aggregation that is also likely. By contrast, other TEIs or dissolved macronutrients may be comparatively unreactive on these time scales and behave quasi-conservatively across the BBL.

To illustrate the application of radiotracers to derive effective vertical fluxes from the ocean's BBL, we consider the early data of Chung & Kim [106]. Here we use the contemporary concentration gradients of dissolved PO_4_^−^ within and overlying the BBL of the Arabian Sea (GEOSECS Station 415, 4000–4175 m water depth, figure 8*b*), combined with their estimates of *K*_v_ from ^222^Rn profiles (figure 8*a*), to calculate the benthic flux of PO_4_^−^ escaping the BBL. Using two estimates of eddy diffusivity (*K*_v1_ and *K*_v2_, figure 8*a*) and the corresponding observed depth gradients in [PO_4_^−^], equation (2.8) derives two identical values for the benthic flux of PO_4_^−^ (0.21 µmol m^2^ d^−1^). We find these estimates to be closely matched by PO_4_^−^ fluxes calculated from the pore waters of sites at equivalent water depths in the Arabian Sea (0.28–1.7 µmol m^2^ d^−1^; 4016–4338 m) [109]. It is important to note that the BBL presented here is well characterized due to its substantial thickness and uncommon sample resolution, but this property is highly varied in the ocean [106]. BBLs were not resolved at all within the lower 10 m of the ocean during GEOSECS, where radiotracers and many TEI distributions will reflect their different sensitivities to solute–particulate interactions, but presently remain a mystery.

The large sample volumes required and the need to make measurements immediately after sample collection have long hampered routine measurements of ^222^Rn and other short-lived radioisotopes. A new generation of continuous flow detectors has improved instrument portability, reduced sample volume requirements and increased sample throughput to allow for higher spatial resolution of radioisotope mixing tracers in the ocean [110–112]. Radium measurements have been adopted in some GEOTRACES transects [113], and longer-lived ^228^Ra has now been used effectively to quantify horizontal fluxes of Co, Fe, Mn and Zn from shelf seas to the open ocean [114]. The shortest-lived radium isotopes (^223^Ra, *t*_1/2_ = 11.2 days; ^224^Ra, *t*_1/2_  =  3.6 days) are perhaps ideally suited for estimating *K*_v_ close to the sediment–water boundary [105,113]. In addition, the determination of another radioisotope tracer, actinium-227 (^227^Ac, *t*_1/2_ = 21.77 years), is an integral result of ^223^Ra measurements by delayed coincidence counting. The measurement of these multiple radioisotopes requires no extra sampling or analytical resource, but provides contingent ways to characterize a BBL of uncertain thickness in a single sampling effort, and for extrapolating boundary fluxes across different space and time scales [115]. Herein we propose the use of radioisotopes across the ocean's BBL to determine effective *K*_v_ and to derive TEI fluxes from contemporary depth profiles. Such a flux determination will integrate a larger area of the seafloor than individual pore water or benthic chamber measurements, including all solute transport processes in pore waters, and, most critically of all, it promises to account for benthic scavenging and/or dissolution processes that may modulate TEI fluxes in the BBL.

#### (i) Summarized utility of benthic boundary layer profiles

*Advantages*

— BBL flux measurements will account for scavenging, dissolution and isotopic exchanges in dilute particle suspensions above the sediment–water interface.
— Pore water solute transport driven by sediment biota and bottom currents will be captured by flux measurement.
— Small-scale horizontal flux variations are smoothed out by turbulent mixing in the BBL.
— Short-lived radium and actinium isotopes are already being measured on some GEOTRACES sections.
— Established trace metal clean sampling and analytical protocols developed by GEOTRACES may be extended to water samples collected from the ocean's BBL.

*Limitations*

— Innovative approaches to sample water for radiotracers and dissolved TEIs close to the seafloor are needed: radium isotope analyses may require the use *in situ* pumps for sampling from large seawater volumes (e.g. 20–100 litres).
— Collection of metre-scale depth-resolved samples from the ocean's BBL is not feasible using equipment hung from a ship's wire in most typical sea surface conditions, as near-bottom sampling risks striking wire-hung equipment on the seabed.
— ‘Effective’ vertical TEI fluxes will be influenced by transport along isopycnal surfaces. The use of three-dimensional models may be required to accurately assess the impact of effective vertical fluxes on oceanic TEI distributions.

### (f) Isotopic mass balance of ocean transect data

Advances in clean sample collection and analysis have enabled the measurement of some transition metal isotope ratios in seawater and suspended particles (e.g. Cd, Cu, Fe, Ni and Zn), and in some cases sediment and pore waters during GEOTRACES section cruises and related studies. These findings have opened the door for isotopes to be used as source tracers to evaluate boundary exchanges of transition metals in the oceans at a range of scales. An equation for isotope mass balance can be used to quantify the fraction (*f*) of a metal (Me) present in the ocean that was contributed from different sources (*a*, *b*) with different isotope compositions 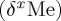:
2.9
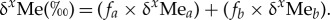

**Figure 9.**
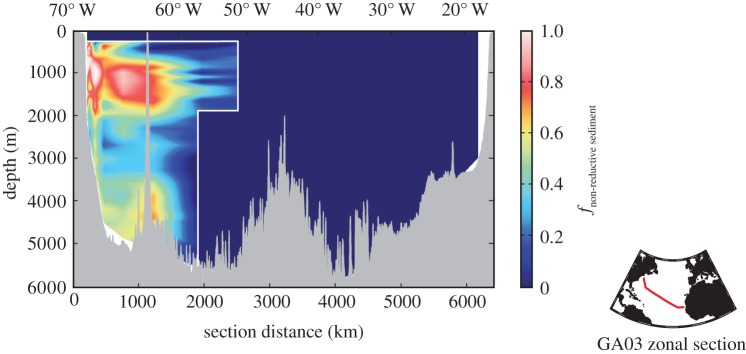
Fraction (*f*) of dissolved Fe in the North Atlantic Ocean sourced from NRD of sediments. Data presented are from the US GEOTRACES GA03 zonal section, and the figure is adapted from Conway & John [116], who used a two-component isotope mixing model to derive *f*, employing pore water and seawater constraints for end-member δ^56^Fe signatures attributed to reductive and non-reductive sediment dissolution [28,29,76].

The isotope ratios of Fe and Zn (δ^56^Fe and δ^66^Zn) have shown great promise in evaluating the sedimentary exchange of these metals at regional and global scales in the ocean. For example, isotopic mass balance has been used to quantify the fractions of dissolved Fe present in the North Atlantic Ocean originating from four discrete sources (atmospheric dust, reductive sediments, non-reductive sediments and hydrothermal vents) based entirely on the measured δ^56^Fe of seawater and knowledge of the end-member isotope compositions [116]. Additionally, *a priori* knowledge of the likely distribution of each source enabled the four-component isotope mixing problem to be broken down into discrete regions where two-component mixing could be used to calculate the influence of each source term within specific regions of the GA03 transect. While these calculations revealed the dominance of the Saharan dust plume for the North Atlantic inventory of dissolved Fe, they also revealed a comparatively minor role for reductive, and major role for non-reductive, sedimentary dissolution of Fe on the western margin (figure 9)—a mechanism of Fe exchange at the sediment–water boundary that was only recently discovered in the ocean [76] and pore waters [29]. The simple model required simplified parameters, so fixed δ^56^Fe end-members for each source were assumed, no appreciable *in situ* fractionation or fractionation during transport was considered, and *a priori* knowledge was used for the allocation of binary sources to discrete regions of the ocean transect. Thus isotope mass-balance models can provide never before seen detail in an ocean basin and make quantitative predictions in specific regions that can direct future investigations. It is also possible that such an approach may also be applied to other metals such as Zn in the near future.

**Figure 10:**
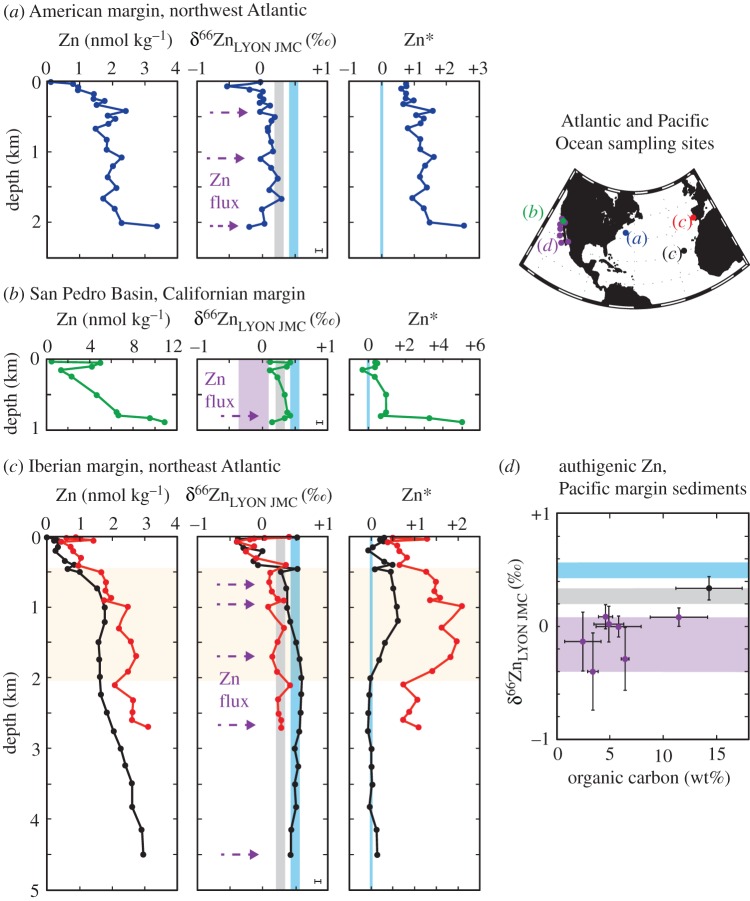
Evidence for benthic fluxes of isotopically light Zn in the water column, and of isotopically light Zn in margin sediments. Water column Zn, δ^66^Zn and Zn* data (*a*–*c*) are reproduced from Conway & John [119,120], and sediment data (*d*) are reproduced from Little *et al*. [118]. The horizontal or vertical light grey or blue bars in isotope plots represent the δ^66^Zn of average deep seawater or lithogenic materials (±1 s.d.) based on Little *et al*. [118], and the purple bars represents the range of authigenic δ^66^Zn measured in Californian and Mexican margin sediments (*d*) [118]. Zn* is the deviation in Zn/Si from deep ocean values after Conway & John [119], with the light blue vertical bars denoting a Zn* based on the deep Atlantic or Pacific Ocean Zn/Si ratio as appropriate. Estimated 2*σ* external uncertainty on water column data (0.05‰) is shown as a single bar in each plot based on replicate analyses of Zn seawater samples (T. M. Conway, 2016, unpublished data).

In contrast with Fe, the cycling of Zn appears to be ‘simpler’. With a residence time (8–17 kyears [117]) exceeding the time scale for ocean mixing, a congruent relationship to Si and well-constrained δ^66^Zn signatures from lithogenic solids (+0.27‰) and deep ocean water (+0.49‰) [118,119], Zn may be better suited to isotope mass-balance calculations of source fractions than Fe. Dissolved Zn with a light isotope composition has been observed in bottom waters and at discrete water depths adjacent to ocean margins (figure 10*a–c*). Light Zn isotope compositions and elevated Zn/Al ratios have also been reported from reducing sediments along the California/Mexico/Peru continental margin, suggesting authigenic enrichments (figure 10*d*) [118]. The elevated Zn concentration and light Zn isotope composition in the water column may record short-term or seasonal events of Zn release at the seafloor, while the sediments record the long-term integrated trend of Zn sequestration into reducing sediments [118,121]. Consequently, the Zn isotope composition of seawater and sediments can be used to constrain Zn exchange between sediments and the water column on various time scales. For example, using the deep ocean Zn/Si relationship (which is different for different ocean basins) and a constant deep ocean δ^66^Zn signature of +0.5‰, Zn isotope mass-balance calculations predict a flux of Zn from sediments that is characterized by a δ^66^Zn signature of −0.5 to −0.8‰ in the Atlantic [119] and −0.3‰ in the San Pedro Basin of the California–Pacific margin [120], consistent with *in situ* sediment measurements from the Californian margin (−0.4 to −0.1‰ [118]; figure 10*d*). At a more regional scale, and using the Zn/Si of deep water in the San Pedro Basin, compared with the deep Pacific, a mass-balance calculation predicts that 40% of dissolved Zn in the basin must originate from sediments [120]. Thus, by combining Zn/Si and δ^66^Zn measurements in this way, it is possible to calculate the proportion of Zn sourced from sediments at discrete locations, and is an approach that could conceivably provide additional insight for other TEIs that bear systematic relationships with macronutrients (e.g. Cd, Cu, Ni).

In summary, when the benthic exchange of a dissolved metal imparts a distinct isotope signature, it may be used to provide a unique quantitative constraint on the impact of that process in the ocean. A key step to improving Zn and probably many other trace metal isotope mass-balance studies is an improved understanding of the isotopic composition of oceanic inputs to, and outputs from, sediments, including the mechanisms by which metal isotopes are sequestered by authigenic mineral phases [117,119,122]. Irrespective of these uncertainties, the cases of Fe and Zn outlined above exemplify the value of simple isotope mass-balance models to derive inventories of TEI exchanges in the ocean at different spatial scales. Further, such models are used to ground-truth isotope palaeoproxies for the reconstruction of past changes in oceanic metal cycles.

To predict how transition metal exchanges at ocean boundaries may change in time and space, we need to improve our knowledge of the metal isotope sequestration pathways in sediments. Such new information may be gained from the study of natural samples and experiments that seek the isotope fractionation attributed to specific mineralization pathways. Enhanced efforts to characterize dissolved and particulate metal isotope partitioning across the sediment–water boundary, with measurements of exchange rate by other methods discussed herein, may yield maximum benefit for the interpretation of TEI exchanges—after which incorporation into computationally more powerful three-dimensional biogeochemical models might also prove feasible and beneficial.

#### (i) Summarized utility of isotopic mass balance

*Advantages*

— Mass-balance calculations use existing and emergent GEOTRACES data products.
— Has the potential to measure the impact of TEI exchange at sediment–water boundary for the oceanic inventory at regional to global scales.
— Can provide information on TEI sources and exchange mechanism.

*Limitations*

— Does not provide a measure of TEI exchanges rates.
— Requires knowledge of the sources and sinks and their end-member isotope compositions.
— Can only distinguish trace element sources bearing distinct isotope signatures.
— Assumes negligible isotope effects result from the internal cycling of TEIs in the ocean, where processes and isotope effects are presently not well constrained.

#### (ii) Biogeochemical models and inverse methods

Three-dimensional global biogeochemical models provide a promising avenue to quantify TEI fluxes based on large-scale tracer distributions, especially given the recent availability of high-resolution transect data from the GEOTRACES programme. Presently, we do not know all the mechanistic controls of TEI fluxes, so global biogeochemical models cannot possibly simulate all the processes that drive TEI exchange across the sediment–water boundary in a mechanistic way [6]. However, like other approaches here, biogeochemical models can provide some insight into the rates of net exchange, where they must rely on a wide variety of rate measurements to guide crude parametrizations of the net fluxes across this boundary [123,124]. In turn, models can predict the TEI distributions that arise when measured fluxes are applied over larger spatial scales, providing an important reality check on our ambitions to extrapolate from comparatively sparse rate measurements in the field.

Efforts to resolve trace metal cycling in global models have largely focused on Fe, because its regulatory impact on primary production moderates the uptake, export and burial of carbon in the oceans, which needs to be accounted for in future climate projections. Current state-of-the-art biogeochemical models couple the benthic source of Fe to organic carbon deposition on the seafloor [75,125] and bottom water oxygenation [126] using empirical relationships [5,6] that might be retuned to reduce model–data misfit. To date, these models have not been successful at reaching a consensus estimate for the global magnitude of this source [20]. A major limitation is that Fe concentrations around continental margins and through the deep ocean are governed not only by sediment dissolution coupled to organic matter oxidation, but also by oxidative weathering processes, by the physico-chemical speciation of Fe, the rate of its scavenging and biological fluxes. These processes are represented very differently between models, if at all, which can therefore arrive at qualitatively similar Fe distributions while predicting benthic sources that vary over orders of magnitude (0.6–155 Gmol yr^−1^ globally [20], §1b, figure 4).

Large-scale TEI distributions place stronger constraints on sedimentary fluxes of elements that (i) undergo less complex chemical speciation and biological cycling than Fe and (ii) have longer residence times that allow the signature of benthic sources/sinks to accumulate over large spatial scales. One example is the distribution of the REEs in seawater. Although fluxes from sediments are not readily observable in section plots of REE concentration, de-convolving the water masses found in such a section, and assessing the expected REE concentration from water mixing, enable deviations to be clearly seen and quantified (e.g. [63,127]), and these deviations to be linked to possible sources from or sinks to marine sediments. This approach has been a notable success of the GEOTRACES programme in helping to assess sedimentary TEI fluxes. It stops short, however, of providing full quantification of these fluxes. To achieve that, inverse modelling methods present powerful but as-yet unexploited tools to extract rate estimates from observed distributions. In its simplest guise, an inverse method is a procedure that adjusts model fluxes, or the parameters controlling them, until the predicted tracer distribution reaches the best possible agreement with observations. It yields more reliable results when larger datasets are available to assess the model prediction, and when *a priori* information of the fluxes and their environmental controls can be replaced by direct rate measurements.

#### (iii) Example: inverse model of Al flux in the North Atlantic Ocean

**Figure 11.**
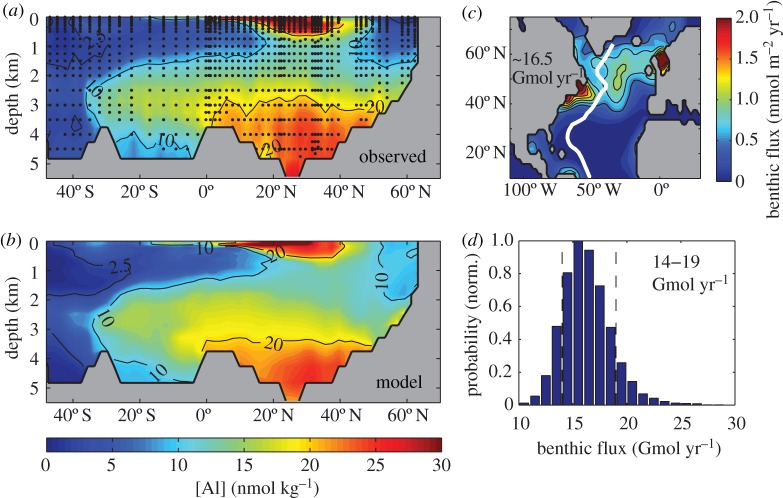
Inverse modelling of benthic Al exchange in the Atlantic Ocean. (*a*) The observed Al distribution along GEOTRACES transect GA02 reveals a tongue of elevated [Al] extending southwards from North Atlantic sediments between 3000 and 4000 m. Black dots indicate data locations used for interpolation by the colour map. (*b*) Simulated Al distribution in our model following parameter optimization, which accurately reproduces the observed large-scale patterns (*R*^2^ = 0.92, RMSE = 1.7 nM). (*c*) Areal rates of benthic Al supply from seafloor sediments in the optimized model. Integrated over North Atlantic (10°–75° N, excluding Mediterranean), a source of 16.5 Gmol(Al) yr^−1^ is most compatible with the observed Al distribution. White line is the cruise track of GA02. (*d*) Probability density function for the basin-wide benthic source, derived by propagating posterior uncertainties in the optimized model-estimated rates. Given the available observations, the basin-wide rate is unlikely to fall outside the range 14–19 Gmol(Al) yr^−1^.

Inverse methods have been successfully used to quantify macronutrient sources and sinks (e.g. [128]), but have not been widely applied to TEIs owing to their sparsely sampled distributions prior to the GEOTRACES programme. To demonstrate the potential of inverse methods in the GEOTRACES era, we have performed new simulations for Al, an element that satisfies the criteria outlined in the subsection above. The GEOTRACES west Atlantic transect (GA02, [129]) recently revealed a prominent tongue of dissolved Al apparently emanating from seafloor sediments in the North Atlantic (40°–50° N) at a depth of approximately 4000 m (figure 11*a*), and spreading southwards in North Atlantic Deep Water. This benthic source is understood to result from the resuspension and dissolution of Al that reaches the seafloor adsorbed to sinking biogenic opal [130], a process that is inhibited throughout most of the ocean by high concentrations of Si(OH)_4_ in sediment pore water [131]. Uniquely in the world ocean, the North Atlantic combines a large external supply of Al (from Saharan dust) with abundant biogenic opal during springtime diatom blooms and low bottom water Si(OH)_4_, resulting in efficient resuspension and dissolution of Al [129]. Previous modelling studies showed that accounting for this resuspension source dramatically improves simulated Al along the GA02 transect [132] compared with versions with atmospheric sources only [133].

For this article, we have re-assessed sedimentary Al fluxes using an inverse approach, and adapted the previous Al model [132] into a ‘transport matrix’ framework that allows for highly efficient simulations [134]. The continuity equation for dissolved Al (Al_diss_) can be written:
2.10
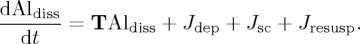

In equation (2.10), **T** (yr^−1^) is the transport matrix that accounts for all physical fluxes (advective and diffusive), and the *J* terms represent sources and sinks of Al (nmol m^−3^ yr^−1^); *J*_dep_ represents the atmospheric source of Al to the surface ocean, computed from previous estimates of dust deposition [135] and a uniform solubility constant; *J*_sc_ represents reversible scavenging, i.e. the net effect of adsorption and desorption to biogenic opal; finally, *J*_resusp_ represents the resuspension flux of Al across the sediment–water interface, controlled by bottom water Si(OH)_4_ and the deposition rate of adsorbed Al onto the seafloor. Given appropriate parametrization of each source and sink (*J* terms, see the electronic supplementary material), equation (2.10) can be solved directly for the steady-state distribution of Al in the Atlantic Ocean, assuming zero net transport across the southern boundary of the basin, where observed concentrations are close to zero (figure 11*a*).

An inverse procedure was used to adjust four key parameters (solubility of Al in dust, scavenging partition coefficient, and two parameters that relate resuspension to Si(OH)_4_) to minimize the misfit between simulated and observed Al (see the electronic supplementary material). The resulting ‘optimum’ model is able to reproduce the GA02 transect data remarkably well (figure 11*b*, *R*^2^ = 0.92, RMSE = 1.7 nM), including the southward-propagating tongue of Al between 3000 and 4000 m. To best reproduce this feature, the optimum model requires a resuspension source of approximately 16.5 Gmol yr^−1^ of Al from seafloor sediments summed across the North Atlantic Ocean (10°–75° N), with the majority occurring in subarctic latitudes polewards of 40° N (figure 11*c*). We used a Monte Carlo approach to propagate ‘posterior’ uncertainty in the model parameters (see the electronic supplementary material) into uncertainty in the model-estimated benthic source, yielding a probability density function for the integrated basin-wide rate (figure 11*d*). This calculation reveals that, assuming the GA02 data accurately capture the time-mean distribution of Atlantic Al, the North Atlantic benthic source probably falls within the range 14–19 Gmol yr^−1^ (1*σ* interval).

Extending this inverse approach globally, and to the other TEIs discussed in this review (e.g. Cd, Cu, Ni and Zn), will require both additional transect data and targeted process studies. While the GEOTRACES programme has achieved sufficient coverage to map large-scale TEI distributions in the Atlantic Ocean, the Pacific Ocean is still sparsely sampled by comparison. Although benthic fluxes of Al are thought to be largest in the Atlantic, the Pacific and Indian Oceans are likely to dominate for other TEI sources, especially those that require strongly reducing sediments. In these cases, existing transect data to date would be insufficient to place robust global bounds on benthic fluxes, especially when direct rate measurements are too sparse to guide model parametrizations. Constraining benthic Fe fluxes remains a unique challenge, but one that must be overcome to improve our predictions of Fe-cycle perturbations. Given the complex biogeochemistry of Fe, it is unlikely that inverse modelling of Fe concentration data alone will suffice, even when data density improves. John *et al*. [68] exemplified the value added from iron isotopes used to constrain a sedimentary end-member in a model of sources and sinks in the San Pedro Basin, California, and studies have shown that different Fe sources leave distinctive isotopic signatures in the water column (e.g. [116], §2f), which might be used as parallel constraints in future models if they prove to be less sensitive to internal Fe-cycle processes in the ocean (e.g particle scavenging/dissolution). Improved use of nephelometry data archives may also be helpful in parametrizing the impacts of scavenging on TEI exchange rates [136]. With these advances, inverse and isotope-resolved models, guided by new rate measurements, offer an exciting (albeit challenging) avenue for evaluating TEI exchanges at the sediment–water boundary.

#### (iv) Summarized utility of biogeochemical and inverse models

*Advantages*

— Models use existing and emerging TEI distribution data in the oceans and, therefore, offer a low-risk (low-cost) approach to evaluate TEI exchange rates.
— Method well suited for conservative TEI tracers.
— Biogeochemical models can test the extrapolation of small-scale rate measurements against large-scale TEI distributions.
— Inverse models extract time-mean fluxes from TEI distributions, integrating across high-frequency variations that might bias individual direct measurements.
— New methods for fast steady-state simulations allow rapid exploration of model ‘parameter space’, placing robust bounds on model-derived rates.
— Relating benthic fluxes to environmental factors (e.g. organic C supply and bottom water O_2_) in biogeochemical models allows us to predict perturbations to TEI cycles under future environmental change.

*Limitations*

— Model fluxes must be parametrized based on direct rate measurements, and thus models compound the limitations inherent to the other methods described herein.
— Mechanisms of sediment–water exchange cannot be represented explicitly due to coarse model resolution, so key environmental sensitivities may be missing.
— Inverse models struggle to disentangle different processes that leave similar signatures in the water column, limiting their application to TEIs with complex biogeochemical cycles such as Fe, unless orthogonal constraints are available.
— Inverse modelling methods require well-sampled tracer distributions, which are presently not available for many TEIs, especially in the Pacific, Southern, Indian and Arctic Oceans.

## 3. Recommendations for future campaigns

In the preceding section, we presented a number of tractable approaches to identify and measure rates of TEI exchange at the sediment–water boundary. None of them, however, come without practical or theoretical limitations to their utility, and all of them will have varying degrees of value and feasibility depending on the time scale, resource, ambition of, and compatibility with, future research. Consequently, optimal strategies to evaluate benthic fluxes may vary between TEIs and need to be designed within individual research proposals. For example, in the case of Fe, pore water data are widely available, but there are very few studies detailing the pore water speciation of Fe and knowledge of the mechanisms of its dissolution and recycling. Estimates of benthic Fe flux that account for scavenging in the bottom boundary layer are critically missing, and a wider range of seafloor conditions, notably more oxic regions, may be useful to refine chamber flux estimates that are used for parametrizations in ocean biogeochemical models. In the case of Zn, such little information exists that a comparatively simple estimate of dissolved concentration gradients in surface sediment pore water is an obvious first-order priority, and making such observations in sediments that receive contrasting amounts of organic carbon or experience contrasting redox conditions would help evaluate the dependence of benthic Zn flux upon such possible parameters. Therefore, in planning future investigations of benthic TEI fluxes, it will be wise to consider: (i) what can be learned from existing data, (ii) how might the outlined approaches provide essential and missing information and (iii) from where is new information a priority to advance our knowledge.

The following recommendations are made for sampling methods and innovations that may accompany GEOTRACES section cruises:

— The application of inverse modelling techniques should be used to maximize the benefits of GEOTRACES data. The presented example of an inverse Al model clearly illustrates existing and emerging opportunities to derive first-order constraints on benthic TEI fluxes from TEI distributions in the ocean and identify regions where sediments are expected to have major influences on these distributions. The results of this can be used to evaluate compatibility between measured and predicted rates of exchange.
— The application of isotopic mass balance has been and should continue to be used to quantify the fractional importance of sediments as benthic sources of Fe and Zn (and any other suitable TEIs) to the water column.
— The collection of intact surface sediments and their pore waters should facilitate a variety of TEI flux determinations. Solid-phase material may be used for authigenic enrichment/depletion studies of TEIs. Presently, there is a paucity of rudimentary information from pore waters on the concentration and physico-chemical speciation of many TEIs that is urgently needed to evaluate fluxes. End-member isotopic constraints from pore waters and authigenic phases are also essential for mass-balance interpretations of the water column. Clean and depth-resolved sediment and pore water samples with volumes suited to isotopic analyses may require sampling innovations. *In situ* pore water sampling methodologies may also be developed to support this goal.
— The design of shipboard and laboratory experiments should be used to reveal the mechanisms of specific solid–colloidal–aqueous TEI transformations and record their isotope effects.

Further recommendations will require repeat site occupations or specialist deployment scenarios, and therefore may be most well suited to process studies, where multiple approaches can usefully be combined:

— We recommend the use of *in situ* benthic incubations to measure TEI fluxes and their adaptations to simulate the influence of particle suspensions in the BBL*. In situ* benthic chambers have a proven track record: they have provided some of our most relied-upon TEI flux determinations (§2b), and are well suited to settings with high sedimentation and biophysical irrigation rates.
— Sediment traps are recommended for the assessment of TEI rain rates in ocean basins in regions dominated by biogenic sedimentation integrated over annual time scales. Substituting the traditional sediment burial rate measurement from sediment cores with a Th-normalized accumulation rate from core tops might provide an improved time scale match between methods used to estimate benthic TEI flux.
— Highly depth-resolved sampling of dissolved and particulate TEIs across the ocean's BBL is recommended to provide new measures of benthic TEI flux based on radiotracer profiles. TEI fluxes from the BBL may provide a complementary space and time resolution for comparison with GEOTRACES section data, which also accounts for the impact of TEI scavenging and dissolution in BBLs. Technological adaptations to routine bottle sampling and *in situ* pumping strategies will be needed to accurately resolve sample volumes across metre-scale chemical gradients of the ocean's BBL.

Such process studies might evaluate fluxes in particular settings. On considering where future efforts to quantify the benthic fluxes of TEIs should be targeted, we collated important properties of the sediment–water boundary that would influence the styles and rates of TEI exchange. We outline these in the following section to assist in planning the locations of future measurements, and to support the long-term development of a broadly distributed and multi-parametric database of benthic TEI fluxes.

### (a) Critical factors for benthic fluxes of trace elements and their isotopes in the oceans

**Figure 12.**
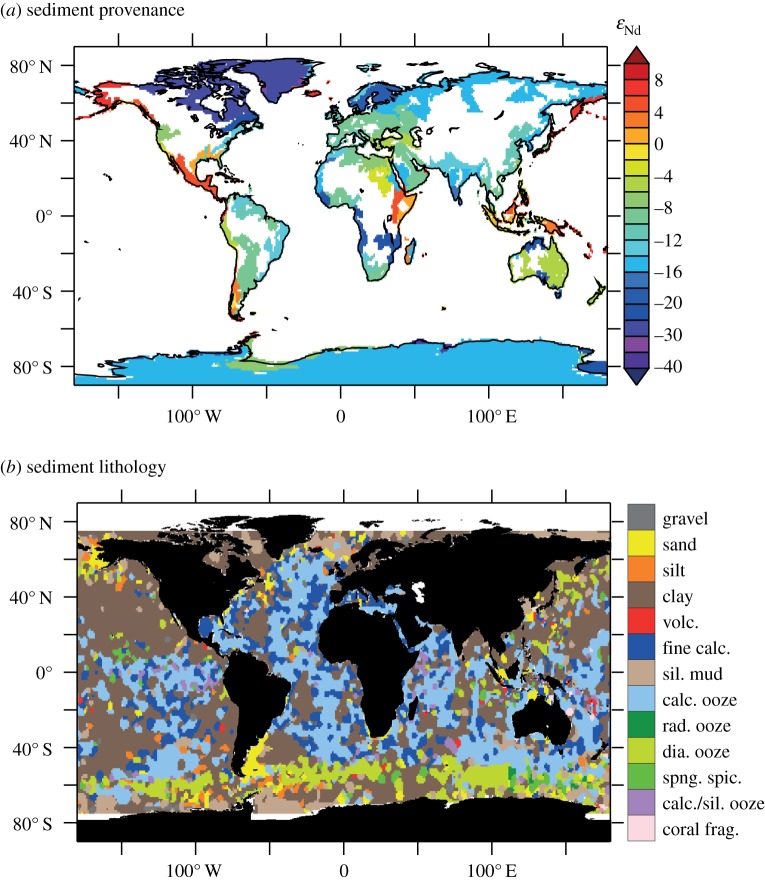

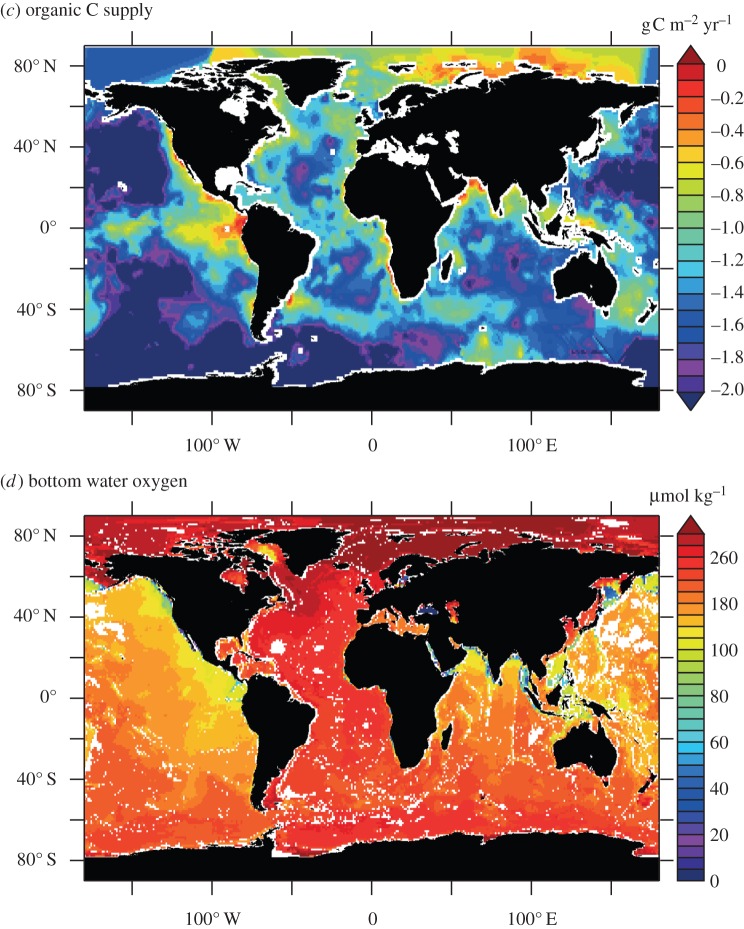

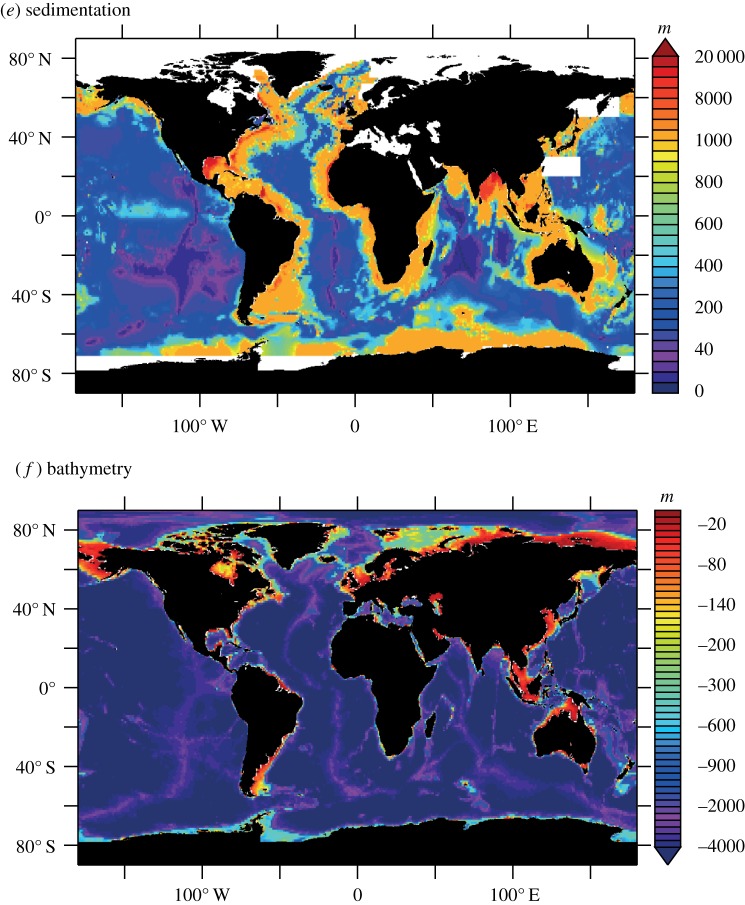

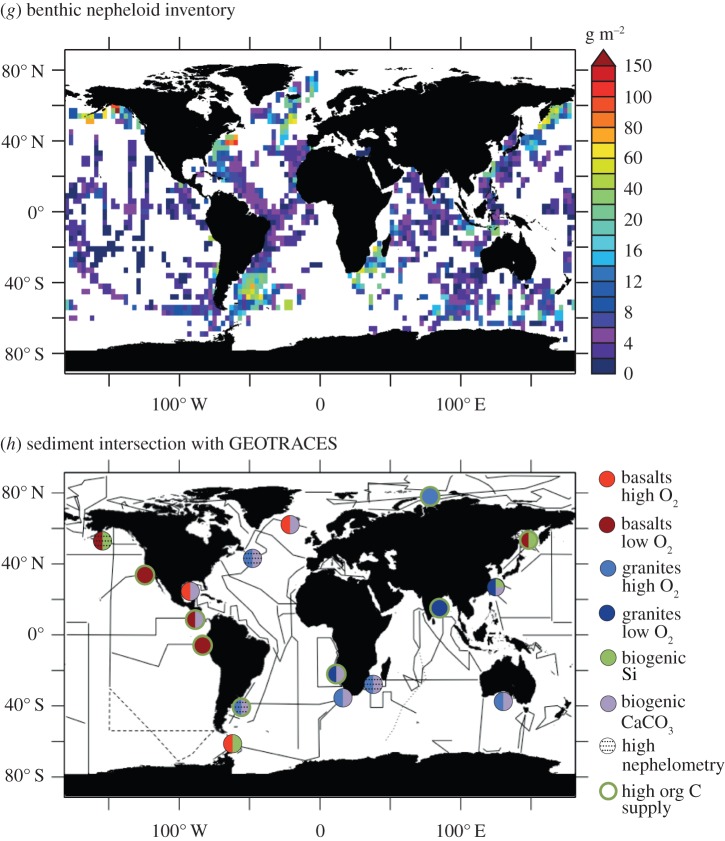
Global distribution of factors considered critical to the benthic exchange of oceanic trace elements and isotopes. (*a*) The Nd isotopic composition (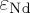) of the continental margins after Jeandel *et al*. [137] is presented here as a geochemical proxy for the provenance of lithogenic material supplied to the adjacent ocean. (*b*) The census of the seafloor after Dutkiewicz *et al*. [138] shows the lithological composition of the seafloor, including lithogenic (gravel, sand, silt, clay, volcanic ash, sand and gravel), biogenic (calcareous ooze, radiolarian ooze, diatom ooze, sponge spicules, mixed calcareous and siliceous ooze, shells and coral fragments) and transitional sediments (fine-grained calcareous sediment, siliceous mud). (*c*) Organic carbon supply to the seafloor described by the data synthesis and calculations of Dunne *et al.* [139]. (*d*) Bottom water oxygen concentration (gridded values within 100 m of the seafloor) is presented from the World Ocean Atlas [140]. (*e*) Seafloor sediment thickness is described by Divins [141]. (*f*) Global mean gridded bathyemtery of the oceans, presented in a nonlinear scale after Amante & Eakins [142]. (*g*) Vertically integrated and gridded benthic nepheloid inventory (g m^−2^) after Biscaye *et al*. [143]. (*h*) An example of ‘SEDITRACES’ sites—where diverse sediment properties described by (*a*–*g*) intersect with the emergent data section lines of the GEOTRACES Science Plan [2].

We shortlist seven criteria that will exert influence over the rates of TEI exchanges and illustrate their oceanographic distribution (figure 12*a–g*), from which we highlight specific regions suited for providing new and critically missing information on TEI exchange rates, where a process study might link with GEOTRACES section data (figure 12*h*). The factors presented are not suggested to account for all TEI flux variability at the sediment–water boundary, nor are they presented in a ranked order of importance (this will probably vary according to individual TEI characteristics), but collectively we consider them to be poorly represented by existing measures of TEI exchange rates.

#### (i) Sediment provenance

Because sediments derived from basalts are more soluble than those derived from granites [23,24], the provenance of lithogenic sediments delivered to the ocean will produce variations to sediment solubility within and between ocean basins. To this end, we consider the 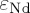 map of continental margins for the global ocean [137] and Mediterranean Sea [144] as a conceptual basis to interpret broad trends in the provenance and solubility of ocean margin sediments (figure 12*a*). Low isotopic compositions (less than −10) correspond to older and more refractory granitic cratons and shales, and higher values (greater than 0) to younger and more easily weathered basaltic terrains. These younger basalts broadly characterize continental margins of the Pacific Ocean, in contrast with other ocean basins. Obvious exceptions are regions of active volcanism, e.g. Gulf of Mexico and Caribbean Sea, Antarctic Peninsula, East Africa, Iceland and volcanic archipelagos, like Kerguelen and the Crozet Islands.

#### (ii) Sediment lithology (including lithogenic versus biogenic)

Ocean sediment itself comprises a mixture of lithogenic and biogenic material inputs in diverse compositions and lithology. A recent census of the seafloor provides a newly revised and detailed approximation of the dominant surface sediment lithology for the global ocean (figure 12*b*) [138]. In regions dominated by biological sedimentation, TEI fluxes are expected to be most sensitive to surface ocean productivity variations [3], whereas in areas dominated by lithogenic material, sediment provenance and physical denudation factors may more strongly exert influence over TEI exchange. Mineral dissolution and authigenesis will also respond to the ecological variance to biogenic Si and biogenic CO_3_ supply terms. In targeting sites for the measurement of lithogenic versus biogenic TEI sources and sinks, seafloor composition must be consulted. Authigenic clay formation, in particular, reverse weathering, is known to play an important role in the sequestration of many major components in seawater (e.g. Na, K and Si) but has probably been overlooked in terms of its significance for TEI budgets [25]. Recent evidence indicates that authigenic clay formation is a significant overlooked sedimentary sink for pore water Fe [145], and elsewhere authigenic Fe- and Mn-bearing clays might also comprise a source of ‘dissolved’ Fe and Mn colloids to bottom waters [26].

#### (iii) Organic carbon supply

The rain of organic carbon that reaches the seafloor is both a carrier of TEIs and the primary energy source for microbial catalysis of many early diagenetic reactions that mobilize and sequester dissolved TEIs in surface sediments. The microbial decomposition of organic matter often exhausts the primary oxidants oxygen and nitrate in surface sediments more rapidly than they are replenished by diffusion from overlying seawater, promoting the onset and extent of Fe and Mn reduction–dissolution. Such oxidant utilization drastically alters the redox environment within surface sediments beneath high-rate organic C supply and promotes the sequestration (e.g. U, Mo, Cd, Zn). The dissolution of Fe–Mn oxides can, in turn, exert control on other trace elements (e.g. Ni [146]) and isotopes (e.g. Tl [147]) via adsorption–desorption processes [79]. Surface ocean primary production principally controls the export of organic C from the surface ocean, and sediments receive that which survives remineralization in the water column (figure 12*c*). Primary production also supplies biogenic minerals (silica, calcite) and their trace element constituents, such that their rain rates will also have some similarities with organic C fluxes at the seafloor. However, organic C supply to the sea floor may not always map closely to primary production rates in the surface water, and thus actual measurements of this process may be necessary to couple with TEI flux measurements. Additionally, the variable reactivity of organic material is likely to drive variable rates of diagenesis, but this is a poorly established parameter.

#### (iv) Bottom water oxygen (redox conditions)

The redox properties of bottom water are of great importance for setting the boundary conditions of the sediment–water interface and controlling the benthic flux of redox-sensitive TEIs. The concentration of oxygen in bottom waters acts in concert with organic carbon supply in determining the depths of Fe and Mn reduction and dissolution and associated TEI cycles. Oxygen and pH in bottom waters further control the solubility and reaction kinetics of redox-sensitive TEIs, such as Fe and Mn, which may escape the sediments [11,148,149]. Figure 12*d* highlights bottom water oxygen as being broadly distinct between Pacific and Atlantic Ocean basins, with most pronounced deficiencies restricted to narrow regions adjacent to the continents (approx. 200–500 m), where sediments intersect permanent oxygen minimum zones [150] that are sensitive to change under the presently warming global climate [151,152]. The redox potential of bottom waters is further related to pH, which is projected to shift to lower values in response to rising atmospheric CO_2_ concentrations [153], with significant impact on, for example, the oxidation kinetics of Fe(ii) and the solubility of many organic and inorganic trace elements in seawater [154,155].

#### (v) Sedimentation

Sediment accumulation rates reflect rates of solid-phase TEI supply and ultimate burial in the ocean. A qualitative measure of this phenomenon is approximated by the thickness of sediments in ocean basins (figure 12*e*). The thickness of sediments is principally controlled by plate tectonics and the age of ocean crust [156], as shown by the contrasting sediment thicknesses of active and passive plate margins. Nevertheless, areas with large volumes of lithogenic material delivered to the continental margin can clearly be identified. An alternative assessment of sedimentation is provided by Archer [157], who estimates the present-day sediment accumulation rates, and finds them to be broadly dominated by pelagic CaCO_3_ sedimentation in central ocean basins above the lysocline. A complexity of this data product for our purposes, however, is that it does not capture the magnitude of lithogenic material passing through the continental shelf and shelf–slope system, where important TEI cycles occur, because much of this delivered material bypasses long-term storage and accumulation in these areas. Indeed, the present thickness of sediment near passive margins is suggested to have been linked to sedimentation pulses resulting from tectonic collisions [156]. Therefore, factors that influence erosion and weathering rates on land in the present day (precipitation patterns, earthquakes and landslides, for example) ought to be considered in a wholistic view of present-day sedimentation in coastal and oceanographic settings.

#### (vi) Bathymetry

The bathymetric depth of the sediment–water boundary will influence many aspects of TEI exchange, which we consider here (figure 12*f*). Seasonality to organic matter input is most strongly expressed in shallower waters, where the impact of TEI exchanges may be closely linked to phytoplankton utilization. Resuspension and physical denudation of sediments may be influenced by wave and bottom current energy. Fine-grained material supplied to ocean margins, and material that undergoes further comminution processes, is preferentially suspended, winnowed and transported to the deep ocean. Such a depth distribution to grain size gives rise to a correlative increase in the reactive surface area for TEI exchanges in deeper parts of the ocean, and maintains broadly contrasting sandy–advective and muddy–diffusive physical characteristics between respective shallow and deep water sediments. The nonlinear bathymetric scale presented in figure 12*f* highlights the contrasting shelf-to-basin ratios of Arctic and Pacific Oceans. Similar approaches may be used in deep waters, to seek the locations of volcanic seamounts or canyon systems that channel lithogenic material into deeper waters, and to distinguish abyssal sites of laminar sedimentation for suitable records of particulate rain and burial.

#### (vii) Benthic nepheloid inventory

The presence of suspended particles in the water column emanating from the BBL highlights regions where TEIs will be most strongly influenced by particle dissolution, adsorption–desorption and isotope exchange. Optical transmission measurements can approximate the vertically integrated benthic nepheloid inventory in the water column that mediates the flux of TEIs across the sediment–water boundary. We present the optical transmission data of Biscaye *et al*. [143] (figure 12*g*). However, many more research ships since this data compilation have collated, and continue to collate, optical transmission data, which could radically improve the resolution of this data product. The data treatment is non-trivial, because careful attention is required to distinguish resuspended from sinking particulate transmission anomalies. Furthermore, the data provide only a rudimentary measure of the true particle concentration with no knowledge of its mineralogy or size. Regardless, this information is routinely collected by CTD casts from research ships all around the world, and it is a missed opportunity if we do not collate it and inform our assessments of particulate distribution and TEI sources and sinks in the oceans.

#### (viii) Sediment intersections with GEOTRACES

Finally, we suggest that all future studies of TEI exchanges at the sediment–water boundary consider their relationship to the criteria listed above (figure 12*a–g*). Opportunities to intersect future rate measurements and process studies with the GEOTRACES section data will maximize our ability to learn the behaviour of TEI cycles within the Earth system. Figure 12*h* summarizes a selection of these so-termed ‘SEDITRACES’ opportunities. The sites chosen are not intended to be prescriptive to future work, and, at the scale presented, details of the data in localized regions may be obscured, such that detailed planning should refer to the original data products where appropriate. Nonetheless, we illustrate a degree of the complexity to the sediment–water boundary that we consider necessary for accurate assessments of TEI exchanges, which are presently missing from ocean biogeochemical models and their biological and climatic predictions.

## 4. Conclusion

We have outlined the principal source and sink pathways for dissolved TEIs at the ocean's sediment–water boundary. On considering our present knowledge of the rates and mechanisms of these exchanges, we find the empirical basis needed for suitably accurate simulations in ocean biogeochemical models to be vital missing knowledge, preventing accurate assessments of the ocean's biological and climatic functions. In the case of Fe, a TEI for which we have arguably made the most determinations of its exchange rates and mechanisms in recent decades, much of our uncertainty concerns the mediatory role of particle scavenging in BBLs, of the physico-chemical speciation of the dissolved forms of Fe, and the exchanges of Fe that occur independently of organic carbon-driven reductive dissolution. We suggest that the lessons learned from Fe must be applied to all TEIs of interest to accelerate new knowledge in these areas.

We have outlined a variety of techniques that we consider tractable ways to identify and measure the rates of TEI exchange at the sediment–water boundary. Each approach offers unique advantages and limitations over the others, and we encourage future field campaigns to consider combinations of these methods to provide the most beneficial results. The majority of modifications to sampling concern the collection of appropriate sample volume and cleanliness for TEI determinations. For every method discussed, an improvement to the determinant of rate will be achieved by careful consideration of the duration/distance across which the TEI exchange is observed, and the temporal/spatial resolution with which the observations are made. For some TEIs, the necessary scales may be achieved with existing sampling technology (e.g. sediment coring and sediment traps); in other cases, new approaches to sample the water column may be required to provide the necessary sample volumes and distributions (e.g. TEIs, particles and radiotracers within BBLs). We consider the use of isotopic mass balance and biogeochemical models themselves to be powerful allies in evaluating the location and magnitude of TEI exchanges, and have exemplified their use with a new inverse model of GEOTRACES section data that allowed us to quantify a major sedimentary source of dissolved Al to the North Atlantic Ocean of approximately 16.5 Gmol yr^−1^.

Finally, we have shortlisted seven criteria that we consider to be of broad relevance to the rates and mechanisms by which TEIs are exchanged at the sediment–water boundary. We have illustrated the distributions of these variable states in the global ocean, and used them to indicate where diverse properties of the sediment–water boundary intersect the GEOTRACES Science Plan, and motivate potential study sites. We suggest that these criteria should underpin the planning of future process studies, so new rate measurements and process understanding can be generated in concert with our advancing views of trace element and isotope distributions. The result of all this will be a more accurate assessment of the biological and climatic impacts of trace elements in the global ocean.

## Supplementary Material

Quantifying trace element and isotope fluxes at the ocean-sediment boundary - supplementary material

## Supplementary Material

README

## Supplementary Material

Aluminium model output

## Data accessibility

The datasets supporting this article have been uploaded as part of the electronic supplementary material.
