# Supplementary material for: Quantifying trace element and isotope fluxes at the ocean–sediment boundary: a review
Source: Philos Trans A Math Phys Eng Sci. 2016 Nov 28;374(2081):20160246. doi: 10.1098/rsta.2016.0246 (PMC5069539; doi:10.1098/rsta.2016.0246)
Supplement: Quantifying trace element and isotope fluxes at the ocean-sediment boundary - supplementary material [file rsta20160246supp1.docx]

Quantifying trace element and isotope fluxes at the ocean-sediment boundary – supplementary material

**William B. Homoky**^1^***, Thomas Weber^2^, William M. Berelson ^3^, Tim M. Conway ^4,5^, Gideon M. Henderson^1^, Marco van Hulten^6^, Catherine Jeandel^7^, Silke Severmann^8^ and Alessandro Tagliabue^9^**

*1. Department of Earth Sciences, University of Oxford, South Parks Road, Oxford, OX1 3AN, United Kingdom (orcid.org/0000-0002-9562-8591)*

*2. School of Oceanography, University of Washington, 1503 NE Boat Street, Seattle, WA  98105, USA*

*3.* *Department of Earth Sciences, University of Southern California, Los Angeles, CA, 90089, USA*

*4. Department of Earth Sciences, ETH Zürich, Clausiusstrasse 25, Zürich, 8092, Switzerland*

*5. College of Marine Science, University of South Florida, St Petersburg, Florida, USA*

*6. Laboratoire des Sciences du Climat et de l'Environnement (LSCE), IPSL, CEA–Orme des Merisiers, 91191 Gif-sur-Yvette, France*

*7. Laboratoire d’Etudes en Géophysique et Océanographie Spatiales (LEGOS), 14 Avenue Edouard* *Belin, 31400, Toulose, France*

*8. Department of Marine and Coastal Sciences, Rutgers University, 71 Dudley Rd., New Brunswick, NJ 08901, USA*

*9. School of Environmental Sciences, University of Liverpool, Jane Herdman Building, Liverpool L69 3GP, United Kingdom*

**Keywords:** GEOTRACES, ocean, sediment, trace element, isotope, benthic boundary layer

*Author for correspondence (will.homoky@earth.ox.ac.uk).

Summary

The document contains supplementary detail of the inverse modelling of aluminium presented and discussed in Section 2.7 of the main article. Including a fuller description of the model equations, and the equations used to optimise the model results and propagate uncertainty.

1. Method of inverse aluminium modelling

1.1 Model description

We simulated the steady-state aluminium (Al) distribution in a three-dimensional ocean circulation model, in which large-scale flow fields are optimized to fit the distributions of passive and transient tracers[1]. This model has a horizontal resolution of 2°x2° latitude/longitude and 24 vertical layers, with vertical resolution ranging from ~30m in the surface and ~500m in the deep ocean. For efficient simulations, we used the Transport Matrix Method[2], in which annual-mean physical fluxes are stored a large sparse matrix (**T**) for offline tracer simulations.

The model domain was confined to the Atlantic Ocean (north of 50°S), where recent GEOTRACES cruises have mapped the distribution of Al, and identified a clear signature of benthic resuspension[3]. This was achieved by dividing the transport matrix into an “interior” component (**T_I_**) that mixes Al within the Atlantic, and a “boundary propagator” component (**T_B_**) that supplies Al across the southern boundary. This method is simplified by the fact that observed Al concentrations are very close to zero at 50°S, meaning that the boundary propagator component can be safely neglected. The continuity equation for Al (*Al_tot_* = dissolved+adsorbed, *Al_diss_*+*Al_ads_* ) can then be written as a combination of physical and biogeochemical fluxes:

$\frac{\partial{Al}_{tot}}{\partial t}=\mathbf{T}_{\mathbf{I}}{Al}_{diss}+J_{dep}+J_{sc}+J_{resusp}$ (Eqn. S1)

Here, *J_dep_* represents the deposition and dissolution of Al in windblown dust, and is represented using a previous model prediction of dust deposition rates (*J_dust_*)[4] and assuming a uniform mass fraction of Al (*f_Al_*) and uniform solubility (*α_Al_*):

$J_{dep}=\alpha_{Al}f_{Al}J_{dust}$ (Eqn. S2)

In all simulations, *f_Al_* was set at 8.2%, and *α_Al_* was treated as a free parameter (see Section 1.2, below).

The adsorption and desorption of Al onto biogenic silica was represented using a reversible scavenging model[5, 6], which captures the net effect of these processes on the distribution of Al through the water column. This assumes that adsorption and desorption reactions proceed much faster than other biogeochemical fluxes (e.g. particle sinking), so that a quasi-steady state is maintained between dissolved and adsorbed (*Al_ads_*) Al:

$\frac{{Al}_{ads}}{{Al}_{tot}}=\frac{K_{sc}pSi}{K_{sc}pSi+1}$ (Eqn. S3)

Here, *K_sc_* is the partition coefficient (ratio of first-order adsorption and desorption rates) and *pSi* represents the concentration of particulate silica (i.e. biogenic opal) available for Al to adsorb onto. Because our model does not contain a prognostic Si cycle, we prescribed the *pSi* distribution based on results of the GFDL Earth System Model v2[7]. The net source or sink of *Al_tot_* at each grid point due to reversible scavenging is then equal to the sinking flux divergence of *Al_ads_*:

$J_{sc}=-\frac{\partial}{\partial z}w_{s}{Al}_{ads}$ (Eqn. S4)

In Eqn. S4, *w_s_* is the sinking velocity of *pSi* onto which Al is adsorbed, taken as 50m/day in our simulations. This formulation of reversible scavenging predicts a net sink of *Al_tot_* near the surface, where scavenging particles are abundant and Al is supplied from the atmosphere, and a net source as the flux of *pSi* attenuates over depth.

In the bottom grid cell of each water column, adsorbed Al is deposited onto the seafloor along with opal that carries it. Of the deposited Al, one fraction is buried in the sediments and lost from the ocean as a whole (balancing the atmospheric source), and the remaining fraction (*f_resusp_*) is resuspended into the overlying water column, constituting a source of *Al_diss_* across the sediment-water interface:

$J_{resusp}={f_{resusp}(w_{s}{Al}_{ads})}_{z=z_{sf}}/\delta z$ (Eqn. S5)

where *z*=*z_sf_* denotes that this sinking flux is evaluated at the depth of the seafloor, and *δz* is the thickness of the grid cell directly overlying the seafloor, into which resuspended Al is released. Following a previous modelling study[8], we relate *f_resusp_* to the concentration of dissolved Si (*Si_diss_*) in bottom water according to the power-law relationship:

$f_{resusp}=min(1,A_{si}{{Si}_{diss}}^{b_{si}})$ (Eqn. S6)

Here, the minimum function ensures that the benthic resuspension source cannot exceed the flux of adsorbed Al onto the seafloor.

1.2. Data and optimization

Together, Eqns 1-6 describe a “forward model”, which predicts the Atlantic Al distribution given a set of parameters. We now seek parameter values that bring the model prediction into best agreement with the observed Al distribution along the GA02 GEOTRACES transect. This dataset was downloaded from the GEOTRACES Intermediate Data Product 2014 (http://www.geotraces.org/dp/idp2014) and regridded onto model grid. For each grid cell, we computed the mean (*μ_Al_*) and standard deviation (*σ_Al_*) of all data points that fall within it. For each run of the forward model, the model-data misfit was quantified by a “cost function” defined as:

$cost=\sum_{i} \left( \frac{\left( {Al}_{diss,i}-\mu_{Al} \right)^{2}}{\left( \sigma_{Al}^{2} \right)} \right)$ (Eqn. S7)

Here, the index *i* denotes those model grid cells that contain data.

We used an optimisation procedure (MATLAB *fminsearch*) to find the values of four key parameters (*α_Al_*, *K_sc_*, *A_Si_*, *b_Si_*) that minimise the value of this cost function. Subject to this parameter set, Eqns 2-6 then yield the Al flux rates most compatible with the GA02 dataset, including the benthic resuspension flux that is of particular interest in this work.

1.3. Error propagation

We quantified the uncertainty in benthic fluxes estimated from our model optimization, by propagating the posterior uncertainty in model parameters into the flux calculations. Simply put, when a similar degree of model-data misfit can be achieved over a broader range of parameter values, those parameters (and the resulting fluxes) are more uncertain. To reflect this, we defined a posterior probability density function (P) for the parameter set *p* (*p=*[ *α_Al_*, *K_sc_*, *A_Si_*, *b_Si_*]) as:

$\log_{10}\left( P\left( p \right) \right)=\sum_{i} \left( \frac{\left( {Al}_{diss,i}-\mu_{Al} \right)^{2}}{\left( \sigma_{Al}^{2} \right)} \right)$ (Eqn. S8)

where the right hand side of the equation is identical to the cost function as defined in Eqn. S7 (e.g.[9]). We used our forward model (Eqns S1-6) and the MATLAB function *slicesample* to map out this PDF across a broad range of parameter space. A Monte Carlo method was then used to draw 10,000 sample parameter sets from this PDF and propagate the parameter uncertainty into the simulated fluxes, resulting in the probability distribution for benthic Al release in the North Atlantic (Figure 9d).

References

[1] DeVries, T. & Primeau, F. 2011 Dynamically and Observationally Constrained Estimates of Water-Mass Distributions and Ages in the Global Ocean. *Journal of Physical Oceanography* **41**, 2381-2401. (doi:10.1175/JPO-D-10-05011.1).

[2] Khatiwala, S. 2007 A computational framework for simulation of biogeochemical tracers in the ocean. *Global Biogeochemical Cycles* **21**, n/a-n/a. (doi:10.1029/2007GB002923).

[3] Middag, R., van Hulten, M.M.P., Van Aken, H.M., Rijkenberg, M.J.A., Gerringa, L.J.A., Laan, P. & de Baar, H.J.W. 2015 Dissolved aluminium in the ocean conveyor of the West Atlantic Ocean: Effects of the biological cycle, scavenging, sediment resuspension and hydrography. *Marine Chemistry* **177, Part 1**, 69-86. (doi:[10.1016/j.marchem.2015.02.015](http://dx.doi.org/10.1016/j.marchem.2015.02.015)).

[4] Mahowald, N.M., Muhs, D.R., Levis, S., Rasch, P.J., Yoshioka, M., Zender, C.S. & Luo, C. 2006 Change in atmospheric mineral aerosols in response to climate: Last glacial period, preindustrial, modern, and doubled carbon dioxide climates. (

[5] Bacon, M.P. & Anderson, R.F. 1982 Distribution of thorium isotopes between dissolved and particulate forms in the deep sea. *Journal of Geophysical Research: Oceans* **87**, 2045-2056. (doi:10.1029/JC087iC03p02045).

[6] Honeyman, B.D., Balistrieri, L.S. & Murray, J.W. 1988 Oceanic trace metal scavenging: the importance of particle concentration. *Deep Sea Research Part A. Oceanographic Research Papers* **35**, 227-246. (doi:[10.1016/0198-0149(88)90038-6](http://dx.doi.org/10.1016/0198-0149(88)90038-6)).

[7] Dunne, J.P., John, J.G., Shevliakova, E., Stouffer, R.J., Krasting, J.P., Malyshev, S.L., Milly, P.C.D., Sentman, L.T., Adcroft, A.J., Cooke, W., et al. 2012 GFDL’s ESM2 Global Coupled Climate–Carbon Earth System Models. Part II: Carbon System Formulation and Baseline Simulation Characteristics. *Journal of Climate* **26**, 2247-2267. (doi:10.1175/JCLI-D-12-00150.1).

[8] van Hulten, M.M.P., Sterl, A., Middag, R., de Baar, H.J.W., Gehlen, M., Dutay, J.C. & Tagliabue, A. 2014 On the effects of circulation, sediment resuspension and biological incorporation by diatoms in an ocean model of aluminium*. *Biogeosciences* **11**, 3757-3779. (doi:10.5194/bg-11-3757-2014).

[9] DeVries, T., Deutsch, C., Primeau, F., Chang, B. & Devol, A. 2012 Global rates of water-column denitrification derived from nitrogen gas measurements. *Nature Geosci* **5**, 547-550. (doi:[doi:10.1038/ngeo1515](http://www.nature.com/ngeo/journal/v5/n8/abs/ngeo1515.html#supplementary-information)).
