# Supplementary material for: Quantifying trace element and isotope fluxes at the ocean–sediment boundary: a review
Source: Philos Trans A Math Phys Eng Sci. 2016 Nov 28;374(2081):20160246. doi: 10.1098/rsta.2016.0246 (PMC5069539; doi:10.1098/rsta.2016.0246)
Supplement: README [file rsta20160246supp2.rtf]

Al_model_output_PTA.mat contains output of optimized Aluminum (Al) cycle model presented in Homoky et al (2016), Quantifying trace element and isotope fluxes at the ocean-sediment boundary - a review, Phil. Trans. Royal Soc. A.File contents1. model_grid: data structure containing grid information for the global grid of the Ocean Circulation Inverse Model (DeVries & Primeau 2011, Journal of Physical Oceanography, 41, pp.2381-2401) used for our Al simulations. Contains the fields:	-lon: longitude (degrees East)	-lat: latitude (degrees North)	-depth depth from sea surface (m)	-dx: horizontal width of grid cells in longitudinal direction (m)	-dy: horizontal width of grid cells in latitudinal direction (m)	-dz: vertical thickness of grid cells (m)2. model_output: data structure containing inverse model-predicted distribution of Al and sources of Al to the ocean. Note, the model was regionalized to simulate only the Atlantic ocean, so all concentrations and fluxes outside of the Atlantic are set to Not a Number (NaN). Contains the fields:	-Al: concentration of dissolved Al (nM)	-Jdep: source of dissolved Al from dust deposition at the sea surface (nmol/m2/yr)	-Jsed: source of dissolved Al from sediment resuspension (nmol/m3/yr)	-Jsed_zint: depth-integrated sediment source (nmol/m2/yr)
